# Trinuclear and Tetranuclear Ruthenium Carbonyl Nitrosyls: Oxidation of a Carbonyl Ligand by an Adjacent Nitrosyl Ligand

**DOI:** 10.3390/molecules29174165

**Published:** 2024-09-03

**Authors:** Shengchun Chen, Xuejun Feng, Yaoming Xie, R. Bruce King, Henry F. Schaefer

**Affiliations:** 1 School of Petrochemical Engineering, Changzhou University, Changzhou 213164, China; 2 Department of Chemistry and Center for Computational Quantum Chemistry, University of Georgia, Athens, GA 30602, USA

**Keywords:** trinuclear and tetranuclear carbonyls, ruthenium, density functional theory

## Abstract

Trinuclear and tetranuclear ruthenium carbonyls of the types Ru_3_(CO)*_n_*(NO)_2_, Ru_3_(N)(CO)*_n_*(NO), Ru_3_(N)_2_(CO)*_n_*, Ru_3_(N)(CO)*_n_*(NCO), Ru_3_(CO)*_n_*(NCO)(NO), Ru_4_(N)(CO)*_n_*(NO), Ru_4_(N)(CO)*_n_*(NCO), and Ru_4_(N)_2_(CO)*_n_* related to species observed experimentally in the chemistry of Ru_3_(CO)_10_(µ-NO)_2_ have been investigated using density functional theory. In all cases, the experimentally observed structures have been found to be low-energy structures. The low-energy trinuclear structures typically have a central strongly bent Ru–Ru–Ru chain with terminal CO groups and bridging nitrosyl, isocyanate, and/or nitride ligands across the end of the chain. The low-energy tetranuclear structures typically have a central Ru_4_N unit with terminal CO groups and a non-bonded pair of ruthenium atoms bridged by a nitrosyl or isocyanate group.

## 1. Introduction

**Figure 1:**
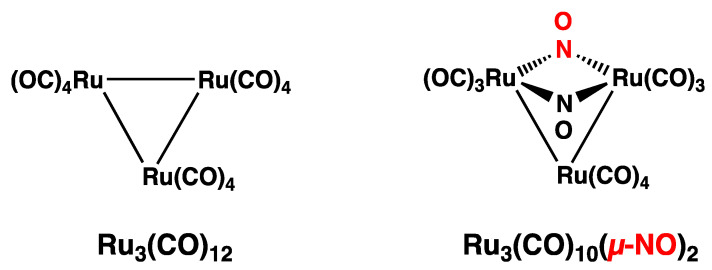
Comparison of the structures of Ru_3_(CO)_12_ and Ru_3_(CO)_10_(µ-NO)_2_.

**Figure 2:**
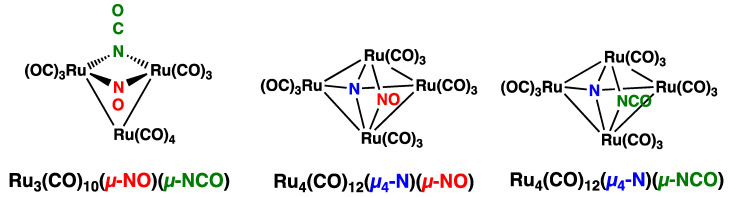
Structure of the decomposition products of Ru_3_(CO)_10_(µ-NO)_2_.

A noteworthy feature of the chemistry of ruthenium is its propensity to form a variety of nitrosyl derivatives. The experimental approach to the subset of such ruthenium nitrosyl derivatives also containing carbonyl ligands starts with the trinuclear derivative Ru_3_(CO)_10_(µ-NO)_2_, itself obtained from the reaction of Ru_3_(CO)_12_ with nitric oxide in boiling benzene (Figure 1) [1]. The replacement of two terminal carbonyl groups in Ru_3_(CO)_12_ with two bridging nitrosyl groups in Ru_3_(CO)_10_(µ-NO)_2_, donating two “extra” electrons to the central Ru_3_ triangle, lengthens one of the three Ru–Ru bonds in the original equilateral triangle Ru_3_(CO)_12_ structure to a non-bonding distance of 3.18 Å. As a result, in the Ru_3_(CO)_10_(µ-NO)_2_ structure, the two nitrosyl groups, as bridges across the non-bonding Ru⋯Ru distance, contribute to holding together the isosceles Ru_3_ triangle. This weaker bonding in the Ru_3_ triangle in Ru_3_(CO)_10_(µ-NO)_2_ relative to that in the Ru_3_ triangle in Ru_3_(CO)_12_ makes the former Ru_3_ triangle more susceptible to rupture and rearrangement. Thus, the decomposition of Ru_3_(CO)_10_(µ-NO)_2_ at 110 °C in an atmosphere of CO leads to the disruption of the Ru_3_ triangle with rearrangement to the tetranuclear derivatives Ru_4_(µ_4_-N)(CO)_12_(µ-NO) and Ru_4_(µ_4_-N)(CO)_12_(µ-NCO), as well as the trinuclear derivative Ru_3_(CO)_10_(µ-NO)(µ-NCO) (Figure 2) [2].

The presence of nitride ligands bridging all four ruthenium atoms in the tetranuclear ruthenium carbonyl nitrosyl derivatives formed in the decomposition of Ru_3_(CO)_10_(µ-NO)_2_ (Figure 2) suggests that the reduction of an NO group by an adjacent CO group is occurring during the decomposition process. Our density functional theory studies of possible internal such redox processes in trinuclear Ru_3_(CO)_10_(µ-NO)_2_ leading to an N_2_O complex Ru_3_(CO)_9_(µ_3_-N_2_O) and finally a dinitrogen complex Ru_3_(CO)_8_(µ_3_-N_2_) were presented in a previous short communication [3]. Here, we present similar density functional theory studies on a wider range of trinuclear and tetranuclear ruthenium carbonyl structures also containing nitrosyl ligands including the reduction of nitrosyl ligands by adjacent CO groups to give, N_2_O and nitride ligands. These include examples of structures with five-electron donor bridging η^2^-µ_3_-NO ligands bonded to ruthenium atoms through both their nitrogen and oxygen atoms as well as structures containing the usual three-electron donor NO groups.

## 2. Results and Discussion

### 2.1. Trinuclear Ru_3_(NO)_2_(CO)_n_ Derivatives

**Figure 3:**
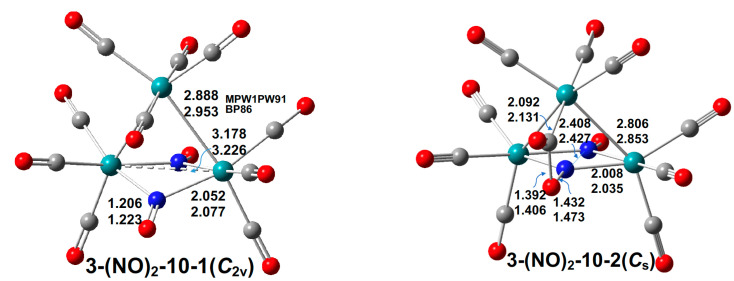
Two optimized Ru_3_(CO)_10_(NO)_2_ structures with bond distances in Å. The upper and lower distances are from the mPW1PW91 and BP86 methods, respectively.

**Figure 4:**
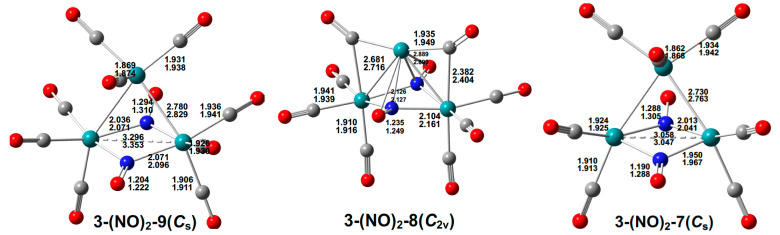
The optimized Ru_3_(CO)*_n_*(NO)_2_ (*n* = 9, 8, 7) structures with bond distances in Å.

**Figure 5:**
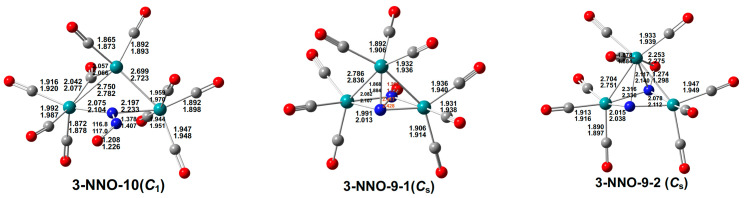
The optimized Ru_3_(N)(CO)_9_(NO) (*n* = 10, 9) structures with bond distances in Å.

**Figure 6:**
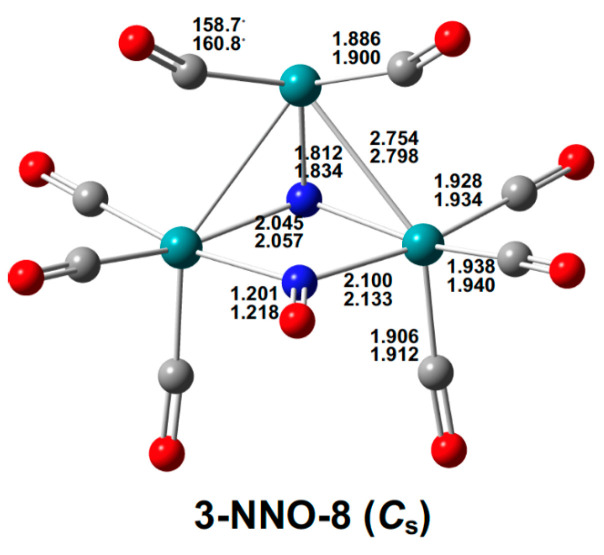
The optimized Ru_3_(N)(CO)_8_(NO) structure with bond distances in Å.

**Figure 7:**
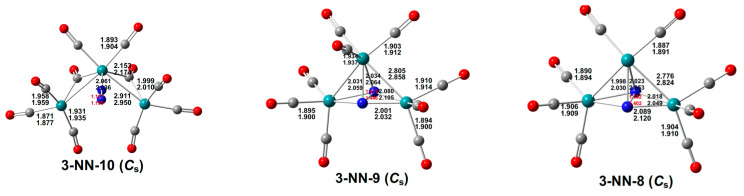
The lowest-energy Ru_3_(N)_2_(CO)*_n_* (*n* = 10, 9, 8) structures with bond distances in Å.

**Figure 8:**
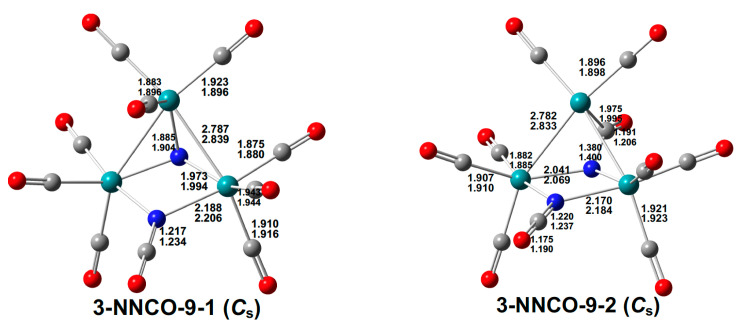
Two isomeric Ru_3_(N)(CO)_9_(NCO) structures with bond distances in Å.

**Figure 9:**
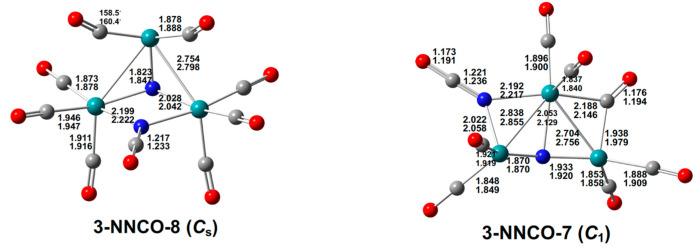
The lowest-energy Ru_3_(N)(NCO)(CO)*_n_* (*n* = 8, 7) structures with bond distances in Å.

**Figure 10:**
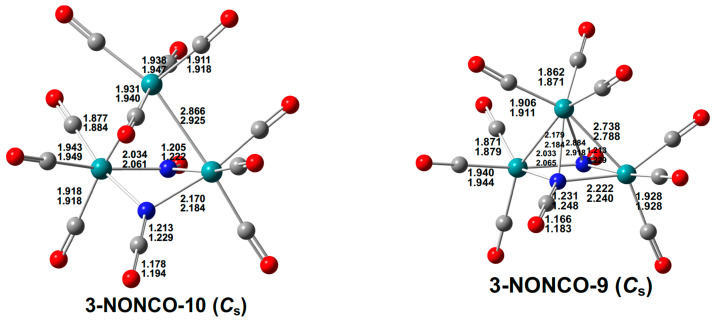
The lowest-energy Ru_3_(CO)*_n_*(NCO)(NO) (*n* = 10, 9) structures with bond distances in Å.

**Figure 11:**
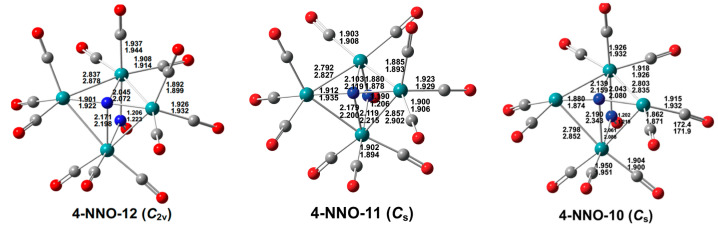
Optimized low-energy Ru_4_(N)(CO)*_n_*(NO) (*n* = 12, 11, 10) structures with bond distances in Å.

**Figure 12:**
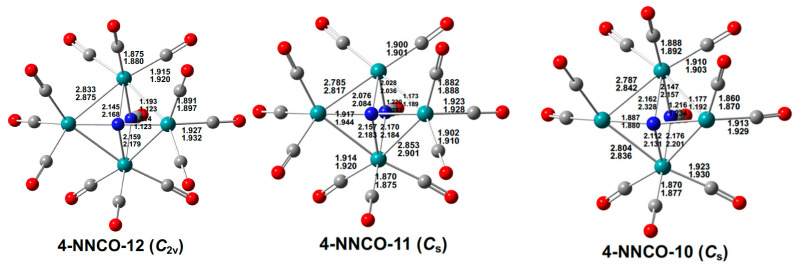
Optimized Ru_4_(N)(CO)*_n_*(NCO) (*n* = 12, 11,10) structures with bond distances in Å.

**Figure 13:**
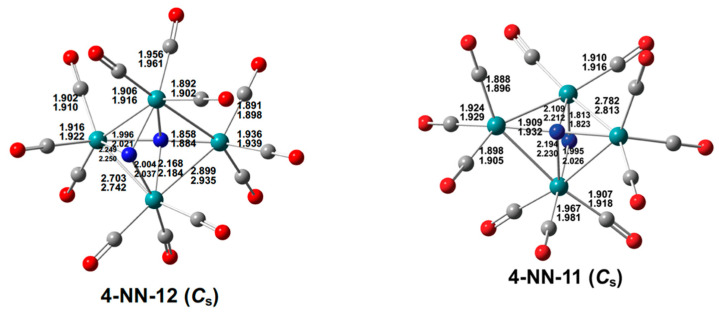
The optimized Ru_4_(N)_2_(CO)*_n_* (*n* = 12, 11) structures with bond distances in Å.

**Figure 14:**
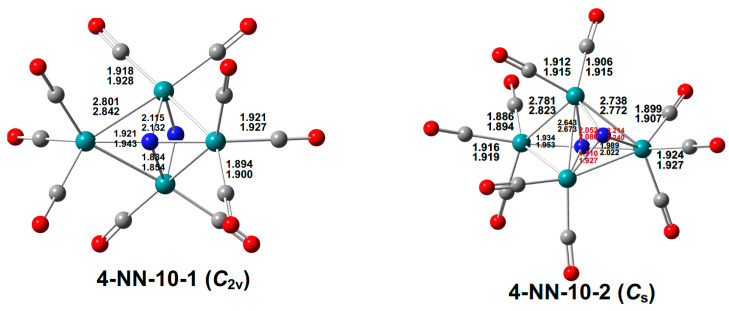
The two Ru_4_(N)_2_(CO)_10_ structures of similar energies with bond distances in Å.

The optimized geometries are depicted in Figure 3, Figure 4, Figure 5, Figure 6, Figure 7, Figure 8, Figure 9, Figure 10, Figure 11, Figure 12, Figure 13 and Figure 14 with all bond lengths in Å. All structures are in singlet state. The structures are designated by the labels **x-A-y-z** or **x-A-y,** where **x** is the number of ruthenium atoms, **A** is the nitrogen-containing group, **y** is the number of carbonyl groups, and **z** orders the isomeric structures (if any) by their relative energies. For example, the singlet global minimum of Ru_3_(CO)_10_(NO)_2_ is designated as **3-(NO)_2_-10-1**.

#### 2.1.1. Ru_3_(CO)_10_(NO)_2_

Two low-energy Ru_3_(CO)_10_(NO)_2_ singlet structures were found (Figure 3). The lowest-energy Ru_3_(CO)_10_(NO)_2_ structure, **3-(NO)_2_-10-1**, is the experimental *C*_2*v*_ structure with two bridging NO groups leading to coplanar Ru_2_NO units. The dihedral angles for the bending of the two Ru_2_N planes in the central Ru_2_(µ-NO)_2_ units in **3-(NO)_2_-10-1** are 156.6° (mPW1PW91) or 157.4° (BP86). The ν(NO) frequencies in **3-(NO)_2_-10-1** are 1557 and 1572 cm^−1^ (BP86) (Table S1 in Supporting Information) as compared with the experimental values [1] of 1500 and 1517 cm^−1^ and consistent with their bridging positions. The Ru–Ru distances of 3.178 Å (mPW1PW91) or 3.226 Å (BP86) indicate no bond between the two ruthenium atoms in the Ru_2_(µ-NO)_2_ units, consistent with the low WBI value of 0.12 (Table S42 in the Supporting Information). The other Ru–Ru distances of 2.888 Å (mPW1PW91) or 2.953 Å (BP86), with WBI values of ~0.3 in the typical range for formal single bonds between d-block metals, correspond to the formal single bond required to give each ruthenium atom the favored 18-electron configuration, since the NO ligands each donate three electrons to the central Ru_2_ unit.

The higher-energy singlet Ru_3_(CO)_10_(NO)_2_ structure **3-(NO)_2_-10-2**, lying 18.4 kcal/mol (mPW1PW91) or 19.1 kcal/mol (BP86) of energy above **3-(NO)_2_-10-1**, has one of the CO groups of its Ru(CO)_4_ unit bent over towards one of the bridging NO groups to form a C–O bond of length 1.392 Å (mPW1PW91) or 1.406 Å (BP86) (Figure 3). This leads to a bridging NOCO group well situated for CO_2_ elimination.^3^ The Ru_2_NO units remain coplanar in **3-(NO)_2_-10-2**. In addition, the entire Ru_2_(µ-NO)_2_ unit in **3-(NO)_2_-10-2** is nearly coplanar as indicated by their dihedral angles of 177.3° (mPW1PW91) or 178.4° (BP86), close to the 180° for ideal planarity. The Ru⋯Ru distances of 3.306 Å (mPW1PW91) or 3.356 Å (BP86), with a low WBI value of 0.08, indicate no direct bond between the two ruthenium atoms in the Ru_2_(µ-NO)_2_ unit. The other Ru–Ru distances of 2.806 Å (mPW1PW91) or 2.853 Å (BP86), with WBI values of ~0.3, correspond to formal single bonds, thereby giving each ruthenium atom the favored 18-electron configuration. The low ν(NO) frequency of 974 cm^−1^ in the NOCO group of **3-(NO)_2_-10-2** is consistent with a single N–O bond rather than the multiple bonds in separate NO ligands. The other ν(NO) frequency in **3-(NO)_2_-10-2** of 1581 cm^−1^ is close to the ν(NO) frequencies of **3-(NO)_2_-10-1** and in a typical region for bridging ν(NO) groups. 

#### 2.1.2. Ru_3_(CO)_n_(NO)_2_ (n = 9, 8, 7)

The single low-energy Ru_3_(CO)*_9_*(NO)_2_ singlet structure **3-(NO)_2_-9** is a *C_s_* structure with two bridging NO groups, leading to essentially coplanar Ru_2_(NO)_2_ units with dihedral angles for the bending of the two Ru_2_N_2_ planes of 175.8° (mPW1PW91) or 176.6° (BP86), close to the 180° indicative of coplanarity (Figure 4). One of the bridging NO groups in **3-(NO)_2_-9** is a five-electron donor η^2^-µ_3_-NO group bonding to two ruthenium atoms through its nitrogen atom with Ru–N distances of 2.036 Å (mPW1PW91) or 2.071 Å (BP86) and to the third ruthenium atom through its oxygen atom with a Ru–O distance of 2.216Å (mPW1PW91) or 2.261 Å (BP86). This η^2^-µ_3_-NO group has a relatively long N–O distance of 1.294 Å (mPW1PW91) or 1.310 Å (BP86), consistent with its very low ν(NO) frequency of 1200 cm^−1^. For comparison, the other NO group in **3-(NO)_2_-9** is a typical three-electron donor bridging µ-NO group with a more typical nitrosyl N–O distance of 1.204 Å (mPW1PW91) or 1.222 Å (BP86) and a more typical bridging ν(NO) frequency of 1571 cm^−1^. The Ru⋯Ru distance of 3.296 Å (mPW1PW91) or 3.353 Å (BP86) in **3-(NO)_2_-9** with a low WBI value of 0.08 similar to that in the Ru_3_(CO)_10_(µ-NO)_2_ structure **3-(NO)_2_-10-1** indicates no direct bond between the two ruthenium atoms in the Ru_2_(NO)_2_ unit. The other Ru–Ru distances of 2.780 Å (mPW1PW91) or 2.829 Å (BP86) with WBI values of ~0.35 correspond to formal single bonds. In **3-(NO)_2_-9**, the combination of two rather than three Ru–Ru bonds in the Ru_3_ triangle, one three-electron donor µ-NO group, one five-electron donor η^2^-µ_3_-NO group, and the nine terminal CO groups give each of the ruthenium atoms the favored 18-electron configuration.

The single low-energy Ru_3_(CO)_8_(NO)_2_ octacarbonyl structure **3-(NO)_2_-8** is a singlet *C_2v_* structure with two µ_3_-NO groups bridging all three ruthenium atoms and two µ-CO groups, each bridging an Ru–Ru bonding edge of length 2.681 Å (mPW1PW91) or 2.716 Å (BP86) with a WBI value of 0.21 (Figure 4). The dihedral angles for the bending of the two Ru_2_N planes in the central Ru_2_(µ_3_-NO)_2_ units in **3-(NO)_2_-8** of 161.2° (mPW1PW91) or 160.8° (BP86) indicate significant deviations from non-planarity. The ν(NO) frequencies of 1435 and 1413 cm^−1^ for the two µ_3_-NO groups bridging all three ruthenium atoms in **3-(NO)_2_-8** are significantly lower than the ν(NO) frequencies of 1572 and 1557 cm^−1^ for the two µ-NO groups bridging Ru–Ru edges in the Ru_3_(CO)_10_(µ-NO)_2_ structure **3-(NO)_2_-10-1**. The two edge-bridging µ-CO groups in **3-(NO)_2_-8** exhibit ν(CO) frequencies of 1907 and 1858 cm^−1^, which are significantly lower than the ν(CO) frequencies of the six terminal CO groups ranging from 2069 to 1983 cm^−1^. The Ru⋯Ru distance of 3.383 Å (mPW1PW91) or 3.490 Å (BP86) in **3-(NO)_2_-8** with a low WBI value of 0.07 indicates no bond between the two ruthenium atoms in the Ru_2_(µ_3_-NO)_2_ units. The other Ru–Ru distances of 2.681 Å (mPW1PW91) or 2.716 Å (BP86) in **3-(NO)_2_-8**, with WBI values of 0.21, correspond to its formal single bonds. This configuration of the Ru–Ru bonds and the bonding of the CO and NO groups to the central Ru_3_ unit in **3-(NO)_2_-8** leads to the favored 18-electron configuration for the two ruthenium atoms in the Ru_2_(µ_3_-NO)_2_ unit, but only a 14-electron configuration for the unique third ruthenium atom. The latter ruthenium atom has a large vacancy in its coordination sphere consistent with its electronic configuration of four electrons less than the favorable 18-electron configuration.

The single low-energy Ru_3_(CO)*_7_*(NO)_2_ heptacarbonyl structure **3-(NO)_2_-7** is a singlet *C_s_* structure with two bridging NO groups (Figure 4). One of the bridging NO groups in **3-(NO)_2_-7** is a five-electron donor η^2^-µ_3_-NO group bonding to two ruthenium atoms through its nitrogen atom with Ru–N distances of 1.950 Å (mPW1PW91) or 1.967 Å (BP86) and to the third ruthenium atom through its oxygen atom with a Ru–O distance of 2.234 Å (mPW1PW91) or 2.281 Å (BP86). This η^2^-µ_3_-NO group has a relatively long N–O distance of 1.288 Å (mPW1PW91) or 1.305 Å (BP86), consistent with its very low ν(NO) frequency of 1220 cm^−1^. The other NO group in **3-(NO)_2_-7** is a typical three-electron donor bridging µ-NO group with an N–O distance of 1.190 Å (mPW1PW91) or 1.288 Å (BP86) and a bridging ν(NO) frequency of 1627 cm^−1^. The dihedral angles for the bending of the two Ru_2_N planes in the central Ru_2_(µ-NO)_2_ units in **3-(NO)_2_-7** are 169.0° (mPW1PW91) or 167.2° (BP86) thereby representing a significant deviation from planarity. The Ru_3_(CO)_7_(µ-NO)(η^2^-µ_3_-NO) structure **3-(NO)_2_-7** can be derived from the Ru_3_(CO)_9_(µ-NO)(η^2^-µ_3_-NO) structure **3-(NO)_2_-9** by the removal of a terminal CO group from each of the ruthenium atoms in the Ru_2_(NO)_2_ unit with relatively little change otherwise in the structure’s geometry. In **3-(NO)_2_-7**, the ruthenium atoms in the Ru_2_(NO)_2_ unit have only a 16-electron configuration, whereas the unique ruthenium atom retains the favorable 18-electron configuration.

### 2.2. Trinuclear Ru_3_(N)(CO)_n_(NO) Derivatives Arising from CO_2_ Loss from Ru_3_(CO)_n_(NO)_2_ Derivatives

The lowest-energy structure **3-NNO-10** of the decacarbonyl is actually an Ru_3_(µ-CO)(CO)_9_(µ-N_2_O) structure with a bent N_2_O ligand bridging the ends of a bent Ru–Ru–Ru chain through Ru–N bonds to its terminal nitrogen atom (Figure 5). One of the carbonyl groups in **3-NNO-10** bridges a Ru–Ru bond in the Ru_3_ chain, exhibiting a ν(CO) frequency of 1929 cm^−1^, significantly lower than any of the terminal ν(CO) frequencies. The bridging bent µ-N_2_O ligand has a single-bond N–N distance of 1.378 Å (mPW1PW91) or 1.407 Å (BP86), a double-bond N=O distance of 1.208 Å (mPW1PW91) or 1.226 Å (BP86), and an N–N–O angle of 116.8°(mPW1PW91) or 117.0° (BP86). This bridging µ-N_2_O ligand functions as a four-electron donor using two lone pairs of the nitrogen atom in the Ru–N–Ru bridge. This gives the two end ruthenium atoms of the Ru–Ru–Ru chain in **3-NNO-10** the favored 18-electron configuration, but the central ruthenium atom only retains a 16-electron configuration. 

The lowest-energy Ru_3_(N)(CO)_9_(NO) structure **3-NNO-9-1** of the nonacarbonyl, like that of the decacarbonyl, is a *C_s_* symmetry structure with a bridging N_2_O group of a different type than that in **3-NNO-10** (Figure 5). Each nitrogen atom in the linear bridging η^2^-µ_3_-N_2_O group of **3-NNO-9-1** forms Ru–N bonds with all three ruthenium atoms. This linear bridging η^2^-µ_3_-N_2_O group has an elongated N–N distance of 2.417 Å (mPW1PW91) or 2.428 Å (BP86) and a double-bond N=O distance of 1.202 Å (mPW1PW91) or 1.220 Å (BP86), and exhibits a ν(NO) frequency of 1579 cm^−1^. The total of six Ru–N bonds formed by the η^2^-µ_3_-NO group allows it to become an 8-electron donor to the Ru_3_ chain, thereby giving each ruthenium atom in **3-NNO-9-1** the favored 18-electron configuration.

As we discussed in Section 2.1.1, CO_2_ elimination may occur from **3-NNO-10-2** to form a higher-energy Ru_3_(N)(CO)_9_(NO) structure **3-NNO-9-2**, which has *C_s_* symmetry with separate N and NO ligands lying 23.3 kcal/mol (mPW1PW91) or 25.4 kcal/mol (BP86) of energy above **3-NNO-9-1** (Figure 5). The NO ligand is a five-electron donor η^2^-µ_3_-NO group bridging all three ruthenium atoms by bonding to two ruthenium atoms through its nitrogen atom with Ru–N distances of 2.316 Å (mPW1PW91) or 2.336 Å (BP86) and to the third ruthenium atom with a Ru–N distance of 2.117 Å (mPW1PW91) or 2.140 Å (BP86) and a Ru–O distance of 2.253 Å (mPW1PW91) or 2.275 Å (BP86). This η^2^-µ_3_-NO group has a relatively long N–O distance of 1.274 Å (mPW1PW91) or 1.298 Å (BP86) consistent with its very low ν(NO) frequency of 1219 cm^−1^. The bridging nitride ligand in **3-NNO-9-2** functions as a three-electron donor by bridging all three nitrogen atoms with two Ru–N distances of 2.015 Å (mPW1PW91) or 2.038 Å (BP86) and one Ru–N distance of 2.316 Å (mPW1PW91) or 2.330 Å (BP86). The combination of a five-electron donor η^2^-µ_3_-NO group, a three-electron donor µ_3_-N nitride ligand, and two Ru–Ru bonds gives each ruthenium atom in **3-NNO-9-2** the favored 18-electron configuration. Compared with the lowest-energy **3-NNO-9-1** structure, **3-NNO-9-2** is relatively unstable towards the elimination of another CO_2_ molecule yielding **3-N_2_-8,** since it has a bent NNO group with its oxygen atom close to the top ruthenium atom.

The lowest-energy octacarbonyl structure Ru_3_(N)(CO)_8_(NO), namely **3-NNO-8**, has separate bridging nitrosyl and nitride ligands (Figure 6). The nitrosyl group bridging two ruthenium atoms with Ru–N distances of 2.100 Å (mPW1PW91) or 2.133 Å (BP86) and a N–O distance of 1.201 Å (mPW1PW91) or 1.218 Å (BP86) exhibits a ν(NO) frequency of 1582 cm^−1^ in a typical region for three-electron donor bridging µ-NO groups. The nitride ligand in **3-NNO-8** bridges all three ruthenium atoms with two Ru–N distances of 2.045 Å (mPW1PW91) or 2.057 Å (BP86) and one Ru–N distance of 1.812 Å (mPW1PW91) or 1.834 Å (BP86). Assigning the favorable 18-electron configuration for the two ruthenium atoms of the Ru_2_(µ_3_-N)(µ-NO) unit leaves a 14-electron configuration for the third ruthenium atom not bonded to the µ-NO group. This is consistent with the apparent gap in its coordination sphere.

### 2.3. Trinuclear Dinitrogen Complexes Ru_3_N_2_(CO)_n_ (n = 10, 9, 8) Arising Formally by Double CO_2_ Loss from Ru_3_(CO)_n_(NO)_2_ Trinuclear Derivatives

The lowest-energy Ru_3_N_2_(CO)_10_ structure **3-NN-10** is a *C_s_* structure having a central bent Ru_3_ unit with Ru–Ru distances of 2.911 Å (mPW1PW91) or 2.950 Å (BP86), corresponding to WBI values of 0.31 and thus a formal single bond and a Ru–Ru–Ru angle of 96.5° (mPW1PW91) or 98.9° (BP86) (Figure 7). Both Ru–Ru bonds are bridged by carbonyl groups exhibiting ν(CO) frequencies of 1864 and 1838 cm^−1^, significantly lower than the ν(CO) frequencies of the eight terminal CO groups in the range from 2066 to 1960 cm^−1^. The central ruthenium atom bears two terminal CO groups and a terminal dinitrogen ligand with a N≡N distance of 1.117 Å (mPW1PW91) or 1.139 Å (BP86), leading to the favored 18-electron configuration after considering the two Ru–Ru bonds, each bridged by a CO group. The terminal ruthenium atoms of the Ru–Ru–Ru chain each bear three terminal CO groups, thereby leading to 16-electron configurations.

The lowest-energy Ru_3_N_2_(CO)_9_ structure **3-NN-9** is a *C_s_* symmetry structure in which the dinitrogen unit bridges all three ruthenium atoms (Figure 7). The N–N distance in **3-NN-9** of 1.417 Å (mPW1PW91) or 1.446 Å (BP86) suggests a formal single bond using only one valence electron from each nitrogen atom. This makes the other eight valence electrons of the N_2_ unit available for donation to the Ru_3_ unit, thereby giving each ruthenium atom in **3-NN-9** the favored 18-electron configuration. The lowest-energy Ru_3_N_2_(CO)_8_ structure **3-NN-8** is similar to that of **3-NN-9**, except for one less CO group on the unique ruthenium atom, thereby giving that ruthenium atom only a 16-electron configuration.

### 2.4. Trinuclear Ruthenium Carbonyl Isocyanates Ru_3_(N)(CO)_n_(NCO) and Ru_3_(CO)_n_(NCO)(NO)

A terminal isocyanate group NCO, considered artificially as a neutral pseudohalogen ligand, is a one-electron donor like the halogens themselves. However, a neutral isocyanate group bridging a pair of metal atoms through its nitrogen atom is a three-electron donor similar to a bridging nitrosyl group. Such bridging isocyanate ligands are predicted consistently in polynuclear ruthenium carbonyl derivatives to exhibit a low ν(CO) frequency in the narrow range of 1310 ± 4 cm^−1^ (BP86). In the chemistry of trinuclear and tetranuclear ruthenium carbonyl isocyanate derivatives, an isocyanate ligand can arise by the carbonylation of a nitride ligand.

Two structures of similar energies were found for the nonacarbonyl Ru_3_(N)(CO)_9_(NCO) (Figure 8). Both structures have bent Ru–Ru–Ru chains. The Ru_3_(µ_3_-N)(CO)_9_(µ-NCO) structure **3-NNCO-9-1** of *C_s_* symmetry has its nitride ligand bridging all three ruthenium atoms with two Ru–N distances of 1.973 Å (mPW1PW91) or 1.994 Å (BP86) and one Ru–N distance of 1.885 Å (mPW1PW91) or 1.904 Å (BP86). The isocyanate ligand with an N=C distance of 1.217 Å (mPW1PW91) or 1.234 Å (BP86) bridges the two ruthenium atoms at each end of the chain with Ru–N distances of 2.188 Å (mPW1PW91) or 2.206 Å (BP86) and exhibits a ν(CO) frequency of 1389 cm^−1^. The nitride ligand, as a five-electron donor gives each ruthenium atom in **3-NNCO-9-1** the favored 18-electron configuration.

The other structure, **3-NNCO-9-2**, of similar energy to **3-NNCO-9-1**, has two isocyanate ligands. It may be regarded as Ru_3_(CO)_8_(µ-NCO)(η^3^-µ_3_-NCO), in which the nitride ligand in **3-NNCO-9-1** has been carbonylated to form a second isocyanate ligand that uses all three of its atoms to bond to all three ruthenium atoms in the cluster (Figure 8). This latter isocyanate group, formally considered as neutral, is a five-electron donor to the Ru_3_ system.

The lowest-energy Ru_3_(N)(CO)_8_(NCO) structure, namely the *C_s_* symmetry structure **3-NNCO-8**, has the isocyanate ligand bridging two ruthenium atoms and the nitride ligand bridging all three ruthenium atoms (Figure 9). Structure **3-NNCO-8** can be derived from the Ru_3_(µ_3_-N)(CO)_9_(µ-NCO) structure **3-NNCO-9-1** (Figure 8) by the loss of a CO group from the ruthenium atom not bridged by the isocyanate ligand. In **3-NNCO-8**, this unique ruthenium atom has only a 16-electron configuration whereas its other two ruthenium atoms have the favored 18-electron configuration. Alternatively, **3-NNCO-8** can be derived from the Ru_3_(µ_3_-N)(CO)_8_(µ-NO) structure **3-NNO-8** (Figure 6) by replacing its three-electron donor bridging NO group with a bridging three-electron donor NCO group.

The lowest-energy Ru_3_(N)(CO)_7_(NCO) structure **3-NNCO-7** has a central bent Ru–Ru–Ru chain with the nitride ligand bridging all three ruthenium atoms (Figure 9). One of the edges of the Ru–Ru–Ru chain is bridged by the isocyanate group and the other edge by a CO group.

The lowest-energy Ru_3_(NO)(CO)_10_(NCO) structure **3-NONCO-10** is closely related to the experimental Ru_3_(CO)_10_(µ-NO)_2_ structure [1] by the replacement of one of the bridging µ-NO groups with a bridging µ-NCO group (Figure 10). The bridging ν(NO) frequency of 1564 cm^−1^ in **3-NONCO-10** is essentially the mean of the bridging ν(NO) frequencies of 1572 and 1557 cm^−1^ in the experimental Ru_3_(µ-NO)_2_(CO)_10_ structure **3-(NO)_2_-10-1**. A Ru_3_(CO)_10_(NCO)(NO) derivative exhibiting a bridging µ(NO) frequency of 1507 cm^−1^ has been observed experimentally as a minor product from the decomposition of Ru_3_(CO)_10_(µ-NO)_2_ at 110 °C under 1 atm CO, but has not been structurally characterized [2].

The lowest-energy Ru_3_(CO)_9_(NCO)(NO) structure **3-NONCO-9** can be derived from the Ru_3_(CO)_10_(µ-NCO)(µ-NO) structure **3-NONCO-10** by the removal of one CO group from the Ru(CO)_4_ unit (Figure 10). The NO group in **3-NONCO-9** remains a three-electron donor, as reflected by its ν(NO) frequency of 1525 cm^−1^, but it bridges all three ruthenium atoms, thereby becoming a five-electron donor through two N→Ru dative bonds and one N–Ru single bond. In this way, each ruthenium atom in **3-NONCO-9** can retain the favored 18-electron configuration.

### 2.5. Tetranuclear Derivatives with Central Ru_4_N Units

The decomposition of Ru_3_(CO)_10_(µ-NO)_2_ at 110 °C under a CO atmosphere yields two tetranuclear products, Ru_4_(µ_4_-N)(CO)_12_(µ-NO) and Ru_4_(µ_4_-N)(CO)_12_(µ-NCO), that have been structurally characterized by X-ray crystallography (Figure 11 and Figure 12) [2]. Both species are found to have a central Ru_4_ butterfly unit capped by the nitrogen atom bridging all four ruthenium atoms. The nitrosyl or isocyanate ligand bridges the body of the butterfly and each ruthenium atom bears three CO terminal groups. Considering the bridging µ_4_-N nitride ligand as a donor of all five of its valence electrons and the bridging η^2^-NO or η^2^-NCO group as a three-electron donor, all four ruthenium atoms have the favored 18-electron configuration in these Ru_4_(µ_4_-N)(CO)_12_(µ-X) derivatives (X = NO, NCO).

Low-energy structures very close to the experimental structures [2] are found for the Ru_4_(N)(CO)_12_(X) derivatives (X = NO, NCO). For the Ru_4_(µ_4_-N)(CO)_12_(µ-NO) structure **4-NNO-12** (Figure 11), the calculated Ru–Ru distances of 2.837 Å (mPW1PW91) or 2.878 Å (BP86) with WBI values of ~0.3 are reasonably close to the experimental distances averaging ~2.82 Å. In addition, the two calculated Ru–N distances of 2.045 and 2.171 Å (mPW1PW91) or 2.012 and 2.188 Å (BP86) for the wingtip ruthenium atoms and 1.901 Å (mPWPW91) or 1.922 Å (BP86) for the body ruthenium atoms are reasonably close to the experimental values of 2.16 Å and 1.90 Å, respectively. Similarly, for the Ru_4_(µ_4_-N)(CO)_12_(µ-NCO) structure **4-NNCO-12** (Figure 12), the calculated Ru–Ru distances of 2.833 Å (mPW1PW91) or 2.875 Å (BP86), likewise with WBI values of ~0.3, are close to the experimental distances averaging ~2.82 Å. In addition, the two calculated Ru–N distances of 2.145 and 1.905 Å (mPW1PW91) or 2.168 and 1.927 Å (BP86) are close to the experimental distances of 2.13 and 1.90 Å, respectively.

The decarbonylation of the Ru_4_(µ_4_-N)(CO)_12_(µ-X) (X = NO, NCO) derivatives preserves the central capped butterfly Ru_4_(µ_4_-N) unit as well as the bridging X group in the low-energy structures (Figure 11 and Figure 12). The electronic configurations of the ruthenium atoms in the original Ru(CO)_3_ moieties in **4-NNO-12** and **4-NNCO-12** losing CO groups go from 18 to 16 in this process.

The low-energy structures of the tetranuclear ruthenium dinitride carbonyls Ru_4_(N)_4_(CO)*_n_* are significantly different from those with nitrosyl or isocyanate groups discussed above. Thus the low-energy Ru_4_(N)_2_(CO)_12_ structure **4-NN-12** has a central Ru_4_ tetragon with one nitride ligand bridging all four ruthenium atoms and the other nitride ligand bridging only three ruthenium atoms (Figure 13). Each ruthenium atom bears three terminal carbonyl groups. If the µ_4_-N ligand contributes all five valence electrons and the µ_3_-N ligand contributes only three of its five valence electrons to the central Ru_4_ network, then each ruthenium atom in **4-NN-12** has the favored 18-electron configuration.

The decarbonylation of the Ru_4_(µ_4_-N)(µ_3_-N)(CO)_12_ structure **4-NN-12** to a Ru_4_(N)_2_(CO)_11_ structure has the effect of forming a new Ru–N bond to the µ_3_-N nitride to give a structure **4-NN-11** in which both nitride ligands bridge the entire Ru_4_ central tetragon (Figure 13). If both µ_4_-N nitride ligands in **4-NN-11** are five-electron donors, then each of the four ruthenium atoms has the favored 18-electron configuration. The central Ru_4_N_2_ unit in **4-NN-11** is a distorted octahedron, with each ruthenium vertex forming two Ru–Ru bonds and two Ru–N bonds and each nitrogen vertex forming four Ru–N bonds. 

Further decarbonylation of Ru_4_(N)_2_(CO)_11_ gives two isomeric decacarbonyls Ru_4_(N)_2_(CO)_10_ having essentially the same energy within the error limits of the calculations (Figure 14). Structure **4-NN-10-1** retains the Ru_4_N_2_ distorted octahedron of **4-NN-11** with both nitride ligands bridging the entire Ru_4_ tetragon. Structure **4-NN-10-2** is a bi-capped tetrahedral Ru_4_N_2_ structure with a central Ru_3_N tetrahedron having a Ru_2_N face capped by the fourth ruthenium atom and a Ru_3_ face capped by the second nitrogen atom. Thus, **4-NN-10-2** has five Ru–Ru bonds, one nitrogen atom bonded to four ruthenium atoms, and the other nitrogen atom bonded to only three ruthenium atoms, whereas **4-NN-10-1** has only four Ru–Ru bonds and both nitrogens bonded to all four ruthenium atoms.

### 2.6. Thermochemistry

Table 1 shows the carbonyl dissociation energies of the Ru_3_(CO)*_n_*(NO)_2_ (*n* = 10, 9, 8) derivatives. The dissociation of a carbonyl ligand is highly endothermic for Ru_3_(CO)*_n_*(NO)_2_ (*n* = 10, 9) but only mildly endothermic for Ru_2_(CO)_8_(NO)_2_. This suggests the viability of the Ru_3_(CO)*_n_*(µ-NO)_2_ (*n* = 10, 9) structures for CO dissociation and the possibility of easy CO dissociation from the Ru_3_(µ-CO)_2_(CO)_6_(µ_3_-NO)_2_ structure. 

Table 2 shows the energies for other types of reactions involving the trinuclear ruthenium carbonyl nitrosyls, including their dissociation into smaller fragments. In general, such processes appear to be highly endothermic, suggesting the viability of the indicated trinuclear species. The one exception is possibly the same Ru_3_(CO)_8_(NO)_2_ (**3-(NO)_2_-8**), being only slightly endothermic toward CO dissociation (Table 1) and also nearly being thermoneutral for dissociation into Ru_2_(CO)_6_ and Ru_2_(CO)_2_(NO)_2_ fragments, with the ruthenium atom in the latter species having the favored 18-electron configuration.

## 3. Theoretical Methods

This study uses two different DFT methods. The first DFT method is the BP86 method, which combines Becke’s 1988 exchange functional (B) with Perdew’s 1986 gradient-corrected correlation functional method (BP86) [4,5]. The second method uses a newer generation functional, mPW1PW91, which combines the modified Perdew–Wang exchange functional with Perdew–Wang’s 91 gradient correlation functional [6]. This functional has been shown to be better for second- and third-row transition-metal compounds [7]. 

The geometries of all structures considered were fully optimized using both the MPW1PW91 and BP86 methods. The vibrational frequencies and the corresponding infrared intensities were determined analytically at the same levels. All of the predicted ν(CO) and ν(NO) frequencies discussed in this paper were obtained from the BP86 method, which were found to be close to the experimental results without scaling factors for the compounds containing transition metals [8]. This concurrence may be accidental, since the theoretical vibrational frequencies predicted by BP86 are harmonic frequencies, whereas the experimental fundamental frequencies are anharmonic. All vibrational frequencies are given in the Supporting Information. The NBO analysis used the same DFT methods to provide information on the WBI values for the Ru–Ru interactions discussed in the manuscript (Table S42 of the Supporting Information) [9]. 

The Stuttgart–Dresden double-ζ (SDD) basis set with an effective core potential (ECP) was used for the ruthenium atoms [10,11]. In this basis set, the 28 core electrons for the ruthenium atoms are replaced by ECP. Such an effective core approximation includes scalar relativistic contributions, which become significant for the heavy transition metal atoms. For the ruthenium atoms, our loosely contracted DZP basis set (14s11p6d/10s8p3d) uses the Wachters primitive set augmented by two sets of p functions and one set of d functions contracted following Hood et al. [12,13]. 

The all-electron double-ζ plus polarization (DZP) basis sets, namely, (9s5p1d/4s2p1d), are used for the carbon, oxygen, and nitrogen atoms. The basis sets are Huzinaga and Dunning’s contracted double-ζ contraction sets [14,15] plus a set of spherical harmonic d polarization functions with the orbital exponents α_d_(C) = 0.75, α_d_(O) = 0.85, and α_d_(N) = 0.80. All of the computations were carried out with the Gaussian 09 program [16], in which the fine grid (75, 302) is the default for the numerical evaluation of the integrals.

## 4. Summary

The experimental Ru_3_(CO)_10_(µ-NO)_2_ structure is shown to be a low-energy structure. The decarbonylation of Ru_3_(CO)_10_(µ-NO) is predicted to convert one of the three-electron donor µ-NO groups into a five-electron donor η^2^-µ_3_-NO group bridging all three ruthenium atoms. Further decarbonylation leads to a low-energy Ru_3_(µ-CO)_2_(CO)_6_(µ_3_-NO)_2_ structure with two three-electron donor µ_3_-NO groups bridging all three ruthenium atoms and two µ-CO groups bridging the two Ru–Ru bonds.

The lowest-energy Ru_3_(N)(CO)_9_(NO) structure obtained by the loss of CO_2_ from Ru_3_(µ-NO)_2_(CO)_10_ has a bridging η^3^-µ_3_-N_2_O ligand but with an elongated N–N bond. A higher-energy Ru_3_(µ_3_-N)(CO)_9_(η^2^-µ-NO) isomer has a nitride ligand bridging all three ruthenium atoms and a five-electron donor η^2^-µ-NO group. The decarbonylation of these structures leads to low-energy Ru_3_(µ_3_N)(CO)*_n_*(µ-NO) (*n* = 8, 7) structures in which the nitride ligand bridges all three ruthenium atoms and the three-electron donor µ-NO ligand bridges only two of the ruthenium atoms.

The loss of two CO_2_ units from Ru_3_(CO)_10_(µ-NO)_2_ leads to a low-energy Ru_3_(CO)_8_(η^2^-µ_3_-N_2_) species in which a dinitrogen ligand with an elongated N–N distance bridges all three ruthenium atoms. This type of bridging dinitrogen ligand is also found in the low-energy carbonyl-richer structure Ru_3_(CO)_9_(η^2^-µ_3_-N_2_).

The carbonylation of a nitride ligand leads to an isocyanate ligand. An isocyanate ligand bridging two metal atoms through its nitrogen atom is a three-electron donor similar to an NO ligand bridging two metal atoms. The experimentally observed Ru_3_(CO)_10_(µ-NCO)(µ-NO), as a minor product from the decomposition of Ru_3_(µ-NO)_2_(CO)_10_, is found to be a low-energy structure. The decarbonylation of Ru_3_(CO)_10_(µ-NCO)(µ-NO) to give Ru_3_(CO)_9_(µ-NCO)(µ-NO) is predicted to preserve the central Ru_2_(µ-NCO)(µ-NO) unit.

The decomposition of Ru_3_(CO)_10_(µ-NO)_2_ is also found to produce the tetranuclear derivatives Ru_4_(µ_4_-N)(CO)_12_(µ-NO) and Ru_4_(µ_4_-N)(CO)_12_(µ-NCO) [2]. These are found to be low-energy structures. A central Ru_4_(µ_4_-N) unit is also found to be the key feature in other low-energy tetranuclear ruthenium carbonyl structures of the type Ru_4_(µ_4_-N)(CO)*_n_*(µ-X) (X = NO, NCO; *n* = 12, 11, 10). The low-energy structure of the tetranuclear ruthenium carbonyl dinitride Ru_4_(µ_4_-N)(µ_3_-N)(CO)_12_ has one nitride ligand bonded to all four ruthenium atoms and the other nitride ligand bonded to only three ruthenium atoms. The decarbonylation of this species gives low-energy Ru_4_(µ_4_-N)_2_(CO)*_n_* (*n* = 11, 10) structures in each of which both nitrogen atoms are bonded to all four ruthenium atoms, leading to a central Ru_4_N_2_ octahedron.

## Figures and Tables

**Table 1:** Energies (ΔE) and free energies (ΔG, at 298 K) in kcal/mol for carbonyl dissociation of Ru_3_(CO)*_n_*(NO)_2_ structures. Both ∆E and ∆G include the zero-point vibrational energy (ZPVE) corrections.

Dissociation Reaction	mPW1PW91		BP86	
	ΔE	ΔG	ΔE	ΔG
Ru_3_(CO)_10_(NO)_2→_Ru_3_(CO)_9_(NO)_2_ + CO	29.2	18.7	29.5	19.1
Ru_3_(CO)_9_(NO)_2→_Ru_3_(CO)_8_(NO)_2_ + CO	73.4	63.0	64.3	55.3
Ru_3_(CO)_8_(NO)_2→_Ru_3_(CO)_7_(NO)_2_ + CO	6.4	−4.4	13.6	2.4

**Table 2:** Energies (ΔE) and free energies (ΔG, at 298K) in kcal/mol for disproportionation and fragmentation processes of the trinuclear ruthenium carbonyl nitrosyls. Both ∆E and ∆G include zero-point vibrational energy (ZPVE) corrections. The Ru_3_(CO)*_n_*(NO)_2_ structures considered in Table 2 are the same as those in Table 1.

Dissociation Reaction	mPW1PW91		BP86	
	ΔE	ΔG	ΔE	ΔG
2Ru_3_(CO)_9_(NO)_2_→Ru_3_(CO)_10_(NO)_2_ + Ru_3_(CO)_8_(NO)_2_	44.2	44.3	34.9	36.2
Ru_3_(CO)_10_(NO)_2_→Ru(NO)_2_(CO)_2_ + Ru_2_(CO)_8_	45.6	28.3	25.9	9.4
Ru_3_(CO)_9_(NO)_2_→Ru(NO)_2_(CO)_2_ +Ru_2_(CO)_7_	44.8	28.7	21.4	5.5
Ru_3_(CO)_8_(NO)_2_→Ru(CO)_2_(NO)_2_ +Ru_2_(CO)_6_	12.8	-3.8	4.4	−11.6
Ru_3_(CO)_10_(NO)_2_→Ru_2_(CO)_5_(NO)_2_ + Ru(CO)_5_	40.9	25.5	23.2	8.3
Ru_3_(CO)_10_(NO)_2_→Ru_2_(CO)_6_(NO)_2_ + Ru(CO)_4_	35.8	20.4	27.3	12.5

## Data Availability

Data are contained within the article and Supplementary Materials.

## Supplementary Materials

The following supporting information can be downloaded at https://www.mdpi.com/article/10.3390/molecules29174165/s1, Table S1: All CO and NO vibrational stretching frequencies (in cm^−1^) and their infrared intensities (in km/mol) for the Ru_3_(CO)*_n_*(NO)_2_ (*n* = 10, 9, 8, 7) structures; Table S2: All CO and NO vibrational stretching frequencies (in cm^−1^) and their infrared intensities (in km/mol) for the Ru_3_(N)(CO)*_n_*(NO) (*n* = 10, 9, 8) structures; Table S3: All CO vibrational stretching frequencies (in cm^−1^) and their infrared intensities (in km/mol) for the Ru_3_(N)_2_(CO)*_n_* (*n* = 10, 9, 8) structures; Table S4: All CO and NO vibrational stretching frequencies (in cm^−1^) and their infrared intensities (in km/mol) for the Ru_3_(N)(CO)*_n_*(NCO) (*n* = 9, 8, 7) structures; Table S5: All CO and NO vibrational stretching frequencies (in cm^−1^) and their infrared intensities (in km/mol) for the Ru_3_(CO)*_n_*(NCO)(NO) (*n* = 10, 9) structures; Table S6: All CO and NO vibrational stretching frequencies (in cm^−1^) and their infrared intensities (in km/mol) for the Ru_4_(N)(CO)*_n_*(NO) (*n* = 12, 11, 10) structures; Table S7: All CO and NO vibrational stretching frequencies (in cm^−1^) and their infrared intensities (in km/mol) for the Ru_4_(N)(CO)*_n_*(NCO) (*n* = 12, 11, 10) structures; Table S8: All CO vibrational stretching frequencies (in cm^−1^) and their infrared intensities (in km/mol) for the Ru_4_(N)_2_(CO)*_n_* (*n* = 12, 11, 10) structures; Tables S9–S13: Optimized geometries for the Ru_3_(CO)*_n_*(NO)_2_ (*n* = 10, 9, 8, 7) structures; Tables S14–S17: Optimized geometries for the Ru_3_(N)(CO)*_n_*(NO) (*n* = 10, 9, 8) structures; Tables S18–S20: Optimized geometries for the Ru_3_(N)_2_(CO)*_n_* (*n* = 10, 9, 8) structures; Tables S21–S24: Optimized geometries for the Ru_3_(N)(CO)*_n_*(NCO) (*n* = 9, 8, 7) structures; Tables S25 and S26: Optimized geometries for the Ru_3_(CO)*_n_*(NCO)(NO)_*n*_ (*n* = 10, 9) structures; Tables S27–S29: Optimized geometries for the Ru_4_(N)(CO)*_n_*(NO) (*n* = 12, 11, 10) structures; Tables S30–S32: Optimized geometries for the Ru_4_(N)(CO)*_n_*(NCO) (*n* = 12, 11, 10) structures; Tables S33–S36: Optimized geometries for the Ru_4_(N)_2_(CO)*_n_* (*n* = 12, 11, 10) structures; complete Gaussian 09 reference [16]; Tables S37–S41: Total energies with ZPVE correction (E in Hartree), Total free energies with ZPVE correction (G in Hartree), numbers of imaginary vibrational frequencies (Nimg) for all the structures; Table S42: Wiberg bond indices (WBI) for the Ru–Ru bonds and natural charges on the ruthenium atoms for all of the structures; complete Gaussian 09 reference [16].
