# Supplementary material for: Trinuclear and Tetranuclear Ruthenium Carbonyl Nitrosyls: Oxidation of a Carbonyl Ligand by an Adjacent Nitrosyl Ligand
Source: Molecules. 2024 Sep 3;29(17):4165. doi: 10.3390/molecules29174165 (PMC11397334; doi:10.3390/molecules29174165)
Supplement: Supplementary file 1 [file molecules-29-04165-s001.zip › molecules-3146048-supplementary.pdf]

Trinuclear and Tetranuclear Ruthenium Carbonyl Nitrosyls: Oxidation of a Carbonyl Ligand by an Adjacent Nitrosyl Ligand

Shengchun Chen [1,*], Xuejun Feng [1,2], Yaoming Xie [2], R. Bruce King [2,*] and Henry F. Schaefer [2]

1 School of Petrochemical Engineering, Changzhou University, Changzhou 213164, China

2 Department of Chemistry and Center for Computational Quantum Chemistry, University of Georgia, Athens, GA 30602, USA

* Correspondence: csc@cczu.edu.cn (S.C.); rbking@uga.edu (R.B.K.)

Supporting Information

Table S1. All CO and NO vibrational stretching frequencies (in cm^{-1}) and their infrared intensities (in km/mol) for the $Ru_3(NO)_2(CO)_n$ (n = 10, 9, 8, 7) structures.
Table S2. All CO and NO vibrational stretching frequencies (in cm^{-1}) and their infrared intensities (in km/mol) for the $Ru_3(N)(NO)(CO)_n$ (n = 10, 9, 8) structures.
Table S3. All CO vibrational stretching frequencies (in cm^{-1}) and their infrared intensities (in km/mol) for the $Ru_3(N)_2(CO)_n$ (n = 10, 9, 8) structures.
Table S4. All CO and NO vibrational stretching frequencies (in cm^{-1}) and their infrared intensities (in km/mol) for the $Ru_3(N)(NCO)(CO)_n$ (n = 9, 8, 7) structures.
Table S5. All CO and NO vibrational stretching frequencies (in cm^{-1}) and their infrared intensities (in km/mol) for the $Ru_3(NO)(NCO)(CO)_n$ (n = 10, 9) structures.
Table S6. All CO and NO vibrational stretching frequencies (in cm^{-1}) and their infrared intensities (in km/mol) for the $Ru_4(N)(NO)(CO)_n$ (n = 12, 11, 10) structures.
Table S7. All CO and NO vibrational stretching frequencies (in cm^{-1}) and their infrared intensities (in km/mol) for the $Ru_4(N)(NCO)(CO)_n$ (n = 12, 11, 10) structures.
Table S8. All CO vibrational stretching frequencies (in cm^{-1}) and their infrared intensities (in km/mol) for the Ru_4 $(N)_2(CO)_n$ (n = 12, 11, 10) structures.
Table S9-S13. The optimized geometries for the $Ru_3(NO)_2(CO)_n$ (n = 10, 9, 8, 7) structures.
Table S14-S17. The optimized geometries for the $Ru_3(N)(NO)(CO)_n$ (n = 10, 9, 8) structures.
Table S18-S20. The optimized geometries for the $Ru_3(N)_2(CO)_n$ (n = 10, 9, 8) structures.
Table S21-S24. The optimized geometries for the $Ru_3(N)(NCO)(CO)_n$ (n = 9, 8, 7) structures.
Table S25-S26. The optimized geometries for the $Ru_3(NO)(NCO)(CO)_n$ (n = 10, 9) structures.
Table S27-S29. The optimized geometries for the $Ru_4(N)(NO)(CO)_n$ (n = 12, 11, 10) structures.
Table S30-S32. The optimized geometries for the $Ru_4(N)(NCO)(CO)_n$ (n = 12, 11, 10) structures.
Table S33-S36. The optimized geometries for the $Ru_4(N)_2(CO)_n$ (n = 12, 11, 10) structures.
Table S37-S41. Total energies with ZPVE correction (E in Hartree), Total free energies with ZPVE correction (G in Hartree), number of imaginary vibrational frequencies (Nimg) for all the structures.
Table S42. Wiberg bond indices (WBI) for the Ru–Ru bonds sand natural charges on the ruthenium atoms for all of the structures.
Complete Gaussian 09 reference (Reference 16)

Table S1. All CO and NO vibrational stretching frequencies (in cm^{-1}) and their infrared intensities (in km/mol) for the $Ru_3(NO)_2(CO)_n$ (n = 10, 9, 8, 7) structures. (Those for NO groups are in italics.)

		**MPW1PW91**		**BP86**				**MPW1PW91**		**BP86**	
**3-(NO)$_2$-10-1**	b_1	*1671*	*(399)*	*1557*	*(360)*	**3-(NO)$_2$-10-2**	a'	*1068*	*(105)*	*974*	*(132)*
	a_1	*1688*	*(154)*	*1572*	*(143)*		a'	*1710*	*(215)*	*1581*	*(217)*
	b_2	2103	(316)	1981	(258)		a'	1869	(502)	1774	(410)
	b_1	2114	(78)	1987	(8)		a''	2119	(127)	1989	(67)
	b_2	2130	(62)	1999	(63)		a'	2120	(180)	1992	(150)
	a_2	2131	(0)	1999	(0)		a''	2130	(232)	2000	(219)
	a_1	2125	(379)	2000	(317)		a'	2134	(617)	2004	(547)
	a_1	2130	(881)	2001	(960)		a''	2146	(4)	2011	(8)
	b_1	2143	(2272)	2012	(1996)		a'	2151	(1520)	2016	(1410)
	a_1	2166	(1355)	2039	(944)		a'	2160	(2187)	2032	(1763)
	b_2	2179	(2112)	2047	(1827)		a''	2192	(1986)	2057	(1696)
	a_1	2214	(51)	2080	(84)		a'	2214	(0)	2076	(3)
**3-(NO)$_2$-9**	a'	*1289*	*(132)*	*1200*	*(118)*	**3-(NO)$_2$-8**	b_1	*1489*	*(715)*	*1413*	*(532)*
	a'	*1698*	*(228)*	*1571*	*(222)*		a_1	*1511*	*(0)*	*1435*	*(1)*
	a''	2104	(216)	1980	(134)		b_2	1978	(853)	1859	(613)
	a'	2108	(179)	1981	(148)		a_1	2029	(1071)	1907	(823)
	a''	2123	(121)	1992	(107)		b_2	2119	(73)	1983	(67)
	a'	2127	(697)	1997	(692)		a_2	2143	(0)	2003	(0)
	a''	2138	(0)	2003	(17)		a_1	2143	(1518)	2006	(822)
	a'	2142	(1657)	2009	(1571)		b_1	2146	(1512)	2007	(1362)
	a'	2154	(2159)	2027	(1614)		b_2	2186	(1994)	2049	(1700)
	a''	2184	(2097)	2051	(1778)		a_1	2209	(252)	2069	(248)
	a'	2208	(2)	2071	(4)						
**3-(NO)$_2$-7**	a'	*1314*	*(152)*	*1220*	*(137)*						
	a'	*1736*	*(339)*	*1627*	*(288)*						
	a'	2093	(315)	1973	(391)						
	a''	2099	(437)	1968	(177)						
	a''	2110	(150)	1980	(309)						
	a'	2115	(1510)	1983	(1276)						
	a'	2139	(2181)	2010	(1638)						
	a''	2142	(2032)	2003	(1975)						
	a'	2180	(163)	2044	(174)						

Table S2. All CO and NO vibrational stretching frequencies (in cm^{-1}) and their infrared intensities (in km/mol) for the $Ru_3(N)(NO)(CO)_n$ (n = 10, 9, 8) structures. (Those for NO groups are in italics.)

		**MPW1PW91**		**BP86**				**MPW1PW91**		**BP86**	
**3-NNO-10**	*a*	*1629*	*(262)*	*1501*	*(234)*	**3-NNO-9-1**	*a'*	*1705*	*(229)*	*1579*	*(232)*
	a	1929	(453)	1832	(352)		a'	2100	(222)	1973	(172)
	a	2087	(450)	1965	(373)		a''	2100	(126)	1972	(116)
	a	2124	(262)	1993	(462)		a''	2115	(117)	1986	(92)
	a	2126	(386)	2001	(341)		a'	2119	(374)	1991	(434)
	a	2135	(1006)	2002	(56)		a''	2128	(7)	1996	(12)
	a	2142	(1009)	2005	(1050)		a'	2132	(2002)	2001	(1789)
	a	2143	(2009)	2014	(2052)		a'	2144	(2515)	2017	(1944)
	a	2152	(539)	2020	(332)		a''	2173	(2270)	2040	(1954)
	a	2187	(1804)	2053	(1565)		a'	2199	(4)	2063	(4)
	a	2214	(120)	2080	(193)						
**3-NNO-9-2**	*a'*	*1352*	*(113)*	*1219*	*(112)*	**3-NNO-8**	*a'*	*1709*	*(228)*	*1582*	*(235)*
	a''	2105	(19)	1978	(33)		a''	1974	(346)	1876	(259)
	a'	2118	(461)	1987	(662)		a'	2002	(1105)	1898	(865)
	a''	2120	(68)	1987	(7)		a''	2120	(82)	1987	(57)
	a'	2126	(945)	1995	(771)		a'	2136	(1173)	2002	(1059)
	a''	2129	(414)	2001	(431)		a''	2137	(0)	2002	(0)
	a'	2139	(1733)	2011	(1352)		a'	2140	(1698)	2006	(1482)
	a'	2167	(1639)	2042	(1268)		a''	2181	(1984)	2047	(1741)
	a''	2176	(2035)	2044	(1701)		a'	2203	(216)	2066	(189)
	a'	2207	(78)	2074	(95)						

Table S3. All CO vibrational stretching frequencies (in cm^{-1}) and their infrared intensities (in km/mol) for the $Ru_3(N)_2(CO)_n$ (n = 10, 9, 8) structures.

		**MPW1PW91**		**BP86**				**MPW1PW91**		**BP86**	
**3-NN-10**	a''	1950	(37)	1838	(22)	**3-NN-9**	a''	2089	(142)	1970	(96)
	a'	1979	(868)	1864	(637)		a'	2094	(295)	1973	(159)
	a''	2085	(30)	1960	(0)		a''	2117	(126)	1988	(106)
	a''	2098	(2024)	1973	(1230)		a'	2122	(331)	1994	(502)
	a'	2107	(336)	1984	(805)		a''	2124	(99)	1995	(112)
	a''	2111	(3)	1986	(103)		a'	2129	(2383)	2000	(2122)
	a'	2119	(972)	1989	(404)		a'	2147	(2359)	2024	(1705)
	a'	2141	(2422)	2016	(1862)		a''	2180	(2124)	2052	(1626)
	a''	2176	(1264)	2044	(1365)		a'	2204	(30)	2071	(17)
	a'	2201	(110)	2066	(1306						
**3-NN-8**	a''	2061	(283)	1946	(253)						
	a'	2092	(623)	1975	(329)						
	a''	2114	(93)	1986	(64)						
	a''	2121	(76)	1991	(43)						
	a'	2123	(1769)	1994	(1501)						
	a'	2131	(2378)	2002	(2140)						
	a''	2176	(2275)	2049	(1750)						
	a'	2199	(84)	2066	(57)						

Table S4. All CO vibrational stretching frequencies (in cm^{-1}) and their infrared intensities (in km/mol) for the $Ru_3(N)(NCO)(CO)_n$ (n = 9, 8, 7) structures.

		MPW1PW91		BP86				MPW1PW91		BP86	
**3-NNCO-9-2**	a'	1385	(8)	1306	(7)	**3-NNCO-9-1**	a'	1389	(5)	1310	(4)
	a"	1936	(586)	1837	(447)		a'	2099	(192)	1972	(137)
	a"	2085	(354)	1961	(337)		a"	2100	(117)	1973	(171)
	a"	2117	(217)	1991	(64)		a"	2119	(141)	1989	(137)
	a'	2121	(100)	1991	(107)		a'	2119	(346)	1991	(492)
	a'	2126	(1648)	1997	(1412)		a"	2125	(85)	1996	(20)
	a"	2130	(55)	1999	(10)		a'	2128	(1866)	2000	(1627)
	a'	2137	(2468)	2005	(2145)		a'	2149	(2367)	2022	(1734)
	a"	2183	(2089)	2055	(1644)		a"	2177	(2063)	2049	(1721)
	a'	2201	(132)	2070	(68)		a'	2199	(111)	2067	(85)
**3-NNCO-8**	a'	1388	(4)	1308	(3)	**3-NNCO-7**	a	1399	(23)	1312	(16)
	a"	1969	(342)	1871	(250)		a	1955	(491)	1842	(363)
	a'	2001	(1046)	1897	(814)		a	2086	(529)	1961	(209)
	a"	2124	(205)	1991	(111)		a	2096	(467)	1966	(869)
	a'	2132	(1135)	2001	(998)		a	2110	(1647)	1986	(1031)
	a"	2137	(10)	2004	(13)		a	2126	(2723)	1996	(2398)
	a'	2142	(1387)	2008	(1280)		a	2141	(1526)	2010	(1287)
	a"	2186	(1826)	2054	(1634)		a	2170	(405)	2041	(472)
	a'	2205	(275)	2071	(197)						

Table S5. All CO and NO vibrational stretching frequencies (in cm^{-1}) and their infrared intensities (in km/mol) for the $Ru_3(NO)(NCO)(CO)_n$ (n = 10, 9) structures. (Those for NO groups are in italics.)

		MPW1PW91		BP86				MPW1PW91		BP86	
**3-NONCO-10**	*a'*	*1676*	*(316)*	*1564*	*(291)*	**3-NONCO-9**	*a'*	*1629*	*(305)*	*1525*	*(284)*
	a'	1398	(2)	1321	(2)		a'	1366	(17)	1284	(8)
	a"	2102	(263)	1980	(251)		a"	2092	(120)	1967	(109)
	a'	2107	(73)	1983	(31)		a'	2097	(95)	1972	(48)
	a'	2124	(892)	1997	(907)		a"	2119	(38)	1989	(74)
	a"	2126	(57)	1999	(22)		a'	2121	(537)	1991	(834)
	a'	2127	(95)	2001	(26)		a"	2122	(105)	1993	(70)
	a"	2132	(41)	2000	(153)		a'	2127	(1641)	1997	(1403)
	a'	2140	(2034)	2011	(1807)		a'	2151	(2161)	2027	(1423)
	a'	2163	(1548)	2038	(1050)		a"	2173	(2153)	2044	(1738)
	a"	2182	(2016)	2052	(1720)		a'	2197	(194)	2065	(137)
	a'	2211	(177)	2080	(157)						

Table S6. All CO and NO vibrational stretching frequencies (in cm^{-1}) and their infrared intensities (in km/mol) for the $Ru_4(N)(NO)(CO)_n$ (n = 12, 11, 10) structures. (Those for NO groups are in italics.)

		MPW1PW91		BP86				MPW1PW91		BP86	
**4-NNO-12**	*a_1*	*1679*	*(261)*	*1559*	*(255)*	**4-NNO-11**	*a'*	*1733*	*(472)*	*1643*	*(424)*
	b_2	2101	(502)	1975	(408)		a"	2093	(129)	1967	(130)
	a_2	2103	(0)	1977	(0)		a'	2102	(222)	1976	(181)
	b_1	2103	(40)	1978	(19)		a"	2103	(1)	1979	(0)
	a_1	2105	(55)	1979	(48)		a'	2110	(518)	1986	(90)
	b_2	2123	(0)	1998	(33)		a"	2113	(95)	1986	(368)
	a_2	2125	(8)	1998	(0)		a'	2123	(643)	1997	(667)
	b_1	2126	(296)	2001	(331)		a"	2125	(241)	2001	(243)
	a_1	2135	(1974)	2006	(1652)		a'	2137	(2745)	2009	(2261)
	a_1	2152	(949)	2028	(811)		a'	2161	(1667)	2036	(1347)
	b_1	2168	(2751)	2040	(2166)		a"	2166	(2645)	2040	(2049)
	b_2	2172	(2516)	2044	(2105)		a'	2200	(155)	2068	(161)
	a_1	2205	(17)	2072	(6)						
**4-NNO-10**	*a'*	*1684*	*(309)*	*1566*	*(312)*						
	a'	2068	(3)	1945	(2)						
	a'	2071	(900)	1947	(652)						
	a'	2099	(822)	1975	(768)						
	a'	2109	(860)	1984	(577)						
	a'	2119	(1205)	1994	(446)						
	a'	2119	(240)	1994	(273)						
	a"	2145	(1827)	2008	(1948)						
	a"	2148	(961)	2013	(938)						
	a"	2164	(1639)	2037	(1475)						
	a"	2205	(475)	2070	(373)						

Table S7. All CO vibrational stretching frequencies (in cm^{-1}) and their infrared intensities (in km/mol) for the $Ru_4(N)(NCO)(CO)_n$ (n = 12, 11, 10) structures.)

		**MPW1PW91**		**BP86**				**MPW1PW91**		**BP86**	
**4-NNCO-12**	a_1	1393	(2)	1315	(2)	**4-NNCO-11**	a'	1390	(2)	1311	(1)
	b_2	2098	(391)	1973	(422)		a''	2083	(157)	1956	(140)
	a_1	2100	(2)	1975	(0)		a'	2098	(97)	1973	(86)
	a_2	2102	(0)	1976	(16)		a''	2098	(11)	1974	(2)
	b_1	2102	(40)	1976	(14)		a'	2107	(438)	1982	(360)
	b_2	2120	(80)	1994	(39)		a''	2111	(37)	1983	(42)
	a_2	2123	(0)	1998	(0)		a'	2115	(810)	1989	(950)
	b_1	2125	(249)	2000	(274)		a''	2120	(213)	1996	(217)
	b_1	2130	(1834)	2003	(1651)		a'	2129	(2279)	2002	(1813)
	a_1	2151	(686)	2028	(509)		a'	2158	(1530)	2034	(1118)
	a_1	2169	(2745)	2040	(2168)		a''	2165	(2676)	2037	(2084)
	b_1	2172	(2436)	2049	(1941)		a'	2196	(375)	2065	(355)
	a_1	2203	(155)	2073	(71)						
**4-NNCO-10**	a'	1391	(2)	1310	(1)						
	a''	2066	(12)	1943	(0)						
	a'	2069	(790)	1946	(600)						
	a''	2097	(523)	1973	(592)						
	a'	2101	(973)	1979	(811)						
	a'	2115	(138)	1989	(93)						
	a''	2116	(1713)	1989	(966)						
	a'	2144	(778)	2112	(1577)						
	a''	2149	(1614)	2112	(726)						
	a'	2160	(1436)	2137	(1264)						
	a'	2206	(542)	2076	(414)						

Table S8. All CO vibrational stretching frequencies (in cm^{-1}) and their infrared intensities (in km/mol) for the $Ru_4(N)_2(CO)_n$ (n = 12, 11, 10) structures.

		MPW1PW91		BP86				MPW1PW91		BP86	
**4-NN-12**	a''	2091	(1)	1967	(0)	**4-NN-11**	a''	2098	(77)	1966	(122)
	a'	2094	(295)	1968	(243)		a'	2103	(289)	1977	(223)
	a''	2109	(9)	1982	(7)		a''	2103	(10)	1979	(3)
	a'	2112	(213)	1985	(190)		a'	2111	(554)	1986	(509)
	a''	2115	(163)	1992	(156)		a''	2116	(68)	1986	(68)
	a'	2127	(592)	2000	(477)		a''	2124	(309)	1997	(430)
	a''	2131	(553)	2001	(423)		a'	2124	(465)	2001	(263)
	a'	2138	(1310)	2009	(1223)		a'	2141	(3034)	2011	(2525)
	a'	2158	(1273)	2033	(964)		a'	2157	(1898)	2031	(1581)
	a''	2168	(2375)	2039	(2014)		a''	2166	(2691)	2038	(2140)
	a'	2178	(2538)	2049	(2070)		a'	2199	(76)	2065	(88)
	a'	2209	(11)	2074	(6)						
**4-NN-10-1**	a_2	2095	(0)	1968	(0)	**4-NN-10-2**	a''	2082	(310)	1960	(154)
	b_2	2101	(40)	1977	(40)		a'	2086	(212)	1963	(178)
	b_1	2106	(51)	1980	(14)		a''	2101	(428)	1978	(47)
	a_1	2111	(417)	1986	(423)		a'	2117	(24)	1989	(120)
	a_2	2113	(0)	1986	(0)		a''	2119	(635)	1992	(748)
	b_2	2119	(496)	1992	(482)		a'	2133	(1009)	2006	(389)
	b_1	2126	(3736)	1999	(3318)		a''	2135	(2777)	2007	(2416)
	a_1	2137	(2231)	2009	(1697)		a'	2139	(1573)	2008	(1710)
	b_2	2164	(2686)	2037	(2105)		a'	2179	(2316)	2049	(1831)
	a_1	2192	(15)	2059	(28)		a'	2203	(112)	2068	(138)

Table S9. The optimized geometry for $Ru_3(NO)_2(CO)_{10}$ structure **3-(NO)$_2$-10-1** (C_{2v}).

mPW1PW91	C	1.93197200	0.00000000	1.47382000
	C	0.00000000	1.52078600	2.74649600
	O	3.07978700	0.00000000	1.44119900
	O	0.00000000	2.39761100	3.49255200
	C	-1.93197200	0.00000000	1.47382000
	O	-3.07978700	0.00000000	1.44119900
	C	1.39331800	2.74845800	-0.14674500
	O	2.38459500	0.00000000	-1.58277400
	O	2.23577700	3.42021400	0.24768400
	C	-1.39331800	2.74845800	-0.14674500
	O	-2.23577700	3.42021400	0.24768400
	Ru	0.00000000	0.00000000	1.59121700
	Ru	0.00000000	1.58899500	-0.82039000
	Ru	0.00000000	-1.58899500	-0.82039000
	C	-1.39331800	-2.74845800	-0.14674500
	C	0.00000000	-2.31998900	-2.58277200
	O	0.00000000	-2.76999500	-3.63918000
	O	-2.23577700	-3.42021400	0.24768400
	N	-1.23076200	0.00000000	-1.23292000
	O	2.23577700	-3.42021400	0.24768400
	O	-2.38459500	0.00000000	-1.58277400
	C	0.00000000	-1.52078600	2.74649600
	C	0.00000000	2.31998900	-2.58277200
	O	0.00000000	-2.39761100	3.49255200
	O	0.00000000	2.76999500	-3.63918000

	C	1.39331800	-2.74845800	-0.14674500
	N	1.23076200	0.00000000	-1.23292000
BP86	C	1.93197200	0.00000000	1.47382000
	C	0.00000000	1.52078600	2.74649600
	O	3.07978700	0.00000000	1.44119900
	O	0.00000000	2.39761100	3.49255200
	C	-1.93197200	0.00000000	1.47382000
	O	-3.07978700	0.00000000	1.44119900
	C	1.39331800	2.74845800	-0.14674500
	O	2.38459500	0.00000000	-1.58277400
	O	2.23577700	3.42021400	0.24768400
	C	-1.39331800	2.74845800	-0.14674500
	O	-2.23577700	3.42021400	0.24768400
	Ru	0.00000000	0.00000000	1.59121700
	Ru	0.00000000	1.58899500	-0.82039000
	Ru	0.00000000	-1.58899500	-0.82039000
	C	-1.39331800	-2.74845800	-0.14674500
	C	0.00000000	-2.31998900	-2.58277200
	O	0.00000000	-2.76999500	-3.63918000
	O	-2.23577700	-3.42021400	0.24768400
	N	-1.23076200	0.00000000	-1.23292000
	O	2.23577700	-3.42021400	0.24768400
	O	-2.38459500	0.00000000	-1.58277400
	C	0.00000000	-1.52078600	2.74649600
	C	0.00000000	2.31998900	-2.58277200
	O	0.00000000	-2.39761100	3.49255200
	O	0.00000000	2.76999500	-3.63918000
	C	1.39331800	-2.74845800	-0.14674500
	N	1.23076200	0.00000000	-1.23292000

Table S10. The optimized geometry for $Ru_3(NO)_2(CO)_{10}$ structure **3-(NO)₂-10-2** (C_s).

mPW1PW91	C	-2.93431900	0.21609200	-1.15499800
	C	-2.11575600	-2.45044800	-0.91312200
	O	-3.68994000	0.80359600	-1.78255800
	O	-2.38671600	-3.44362900	-1.42200600
	C	-2.88408400	-0.96331500	1.36688500
	O	-3.58304300	-1.05612800	2.27178500
	C	2.93432300	0.21615200	-1.15497500
	C	2.11576000	-2.45040200	-0.91316500
	O	3.68994300	0.80367700	-1.78251700
	O	2.38670700	-3.44356900	-1.42208400
	C	2.88413100	-0.96333100	1.36685800
	O	3.58312300	-1.05617800	2.27172900
	N	0.00001400	-1.60220900	0.86921200
	N	0.00000300	-0.13505600	-1.04033400
	O	0.00002000	-2.37363800	1.79056100
	O	0.00000100	0.58702500	-2.27621100
	Ru	-1.65303300	-0.79121400	-0.10848200
	Ru	1.65304600	-0.79119000	-0.10847800
	Ru	-0.00001300	1.43946500	0.29553200
	C	-1.48495600	2.63733400	0.49023500
	C	1.48492800	2.63733300	0.49024000
	C	-0.00002100	0.90698000	2.19309400
	C	-0.00002100	1.87567300	-1.75023600

	O	-2.35575200	3.37734100	0.61009700
	O	-0.00003700	2.82553400	-2.48138900
	O	-0.00003700	0.65835800	3.31442500
	O	2.35572900	3.37732900	0.61013700
BP86	C	-2.97918400	0.19549400	-1.14163400
	C	-2.12516700	-2.47039500	-0.92708300
	O	-3.76276400	0.78189900	-1.77203300
	O	-2.40348100	-3.47560000	-1.45272100
	C	-2.91096000	-0.99654800	1.36837600
	O	-3.62402200	-1.10423700	2.28549100
	C	2.97918800	0.19550200	-1.14163000
	C	2.12519100	-2.47038700	-0.92706100
	O	3.76277000	0.78190400	-1.77203000
	O	2.40352100	-3.47559400	-1.45268700
	C	2.91096100	-0.99652200	1.36838800
	O	3.62402100	-1.10420000	2.28550500
	N	0.00000300	-1.60764100	0.88794200
	N	0.00000200	-0.12627300	-1.03474600
	O	0.00000300	-2.37282400	1.83764600
	O	0.00000800	0.60332900	-2.31485500
	Ru	-1.67821100	-0.80678900	-0.10582200
	Ru	1.67821400	-0.80678000	-0.10581500
	Ru	-0.00000900	1.46466600	0.30045100
	C	-1.49255300	2.66855500	0.47150100
	C	1.49253000	2.66856400	0.47149300
	C	-0.00000400	0.95623000	2.21124100
	C	0.00000200	1.90563500	-1.78437800
	O	-2.37012800	3.42965700	0.59132200
	O	0.00000800	2.86979500	-2.52002800
	O	-0.00000900	0.73023100	3.35616100
	O	2.37009400	3.42968200	0.59129500

Table S11. The optimized geometry for $Ru_3(NO)_2(CO)_9$ structure **3-(NO)$_2$-9** (C_s).

mPW1PW91	C	-0.65999400	1.42793100	2.83525300
	C	-2.47827800	-0.64239100	2.15766700
	O	-0.59668000	2.34951100	3.51663600
	O	-3.53049800	-0.99938200	2.45090500
	C	0.15518500	-1.23757400	2.92951200
	O	0.66728200	-1.93003200	3.68546200
	C	-0.65999400	1.42793100	-2.83525300
	C	-2.47827800	-0.64239100	-2.15766700
	O	-0.59668000	2.34951100	-3.51663600
	O	-3.53049800	-0.99938200	-2.45090500
	C	0.15518500	-1.23757400	-2.92951200
	O	0.66728200	-1.93003200	-3.68546200
	N	-0.22462300	-1.17119400	0.00000000
	N	-1.38238600	0.98323200	0.00000000
	O	0.66215200	-2.11290900	0.00000000
	O	-1.99004000	2.02278800	0.00000000
	Ru	-0.72765700	-0.08689400	1.64775900
	Ru	-0.72765700	-0.08689400	-1.64775900
	Ru	1.51146100	-0.06590600	0.00000000
	C	2.73140700	-0.22481900	1.48831700
	O	3.49378700	-0.27976500	2.34702300
	C	2.73140700	-0.22481900	-1.48831700
	O	3.49378700	-0.27976500	-2.34702300
	C	1.54353200	1.80281100	0.00000000
	O	1.57729400	2.95541000	0.00000000
BP86	C	-0.69046500	1.43338000	2.87138000
	C	-2.49408400	-0.65319700	2.16712000
	O	-0.64292900	2.36731800	3.57001000
	O	-3.56020700	-1.02687100	2.46694600
	C	0.13698800	-1.22198900	2.98110200
	O	0.64555800	-1.91658000	3.76668700
	C	-0.69046500	1.43338000	-2.87138000
	C	-2.49408400	-0.65319700	-2.16712000
	O	-0.64292900	2.36731800	-3.57001000
	O	-3.56020700	-1.02687100	-2.46694600
	C	0.13698800	-1.22198900	-2.98110200
	O	0.64555800	-1.91658000	-3.76668700
	N	-0.21988100	-1.18142800	0.00000000
	N	-1.38042800	0.99875900	0.00000000
	O	0.65351400	-2.15717400	0.00000000
	O	-1.98610700	2.06030500	0.00000000
	Ru	-0.73738800	-0.08151000	1.67650000
	Ru	-0.73738800	-0.08151000	-1.67650000
	Ru	1.54085300	-0.07783700	0.00000000
	C	2.76366400	-0.23765600	1.49456200
	O	3.54962400	-0.28056800	2.35810400
	C	2.76366400	-0.23765600	-1.49456200
	O	3.54962400	-0.28056800	-2.35810400
	C	1.57782000	1.79613600	0.00000000
	O	1.62782500	2.96690900	0.00000000

Table S12. The optimized geometry for $Ru_3(NO)_2(CO)_8$ structure **3-(NO)₂-8** (C_{2v}).

mPW1PW91	C	1.41793600	3.01606600	-0.36955500
	C	0.00000000	1.61899800	-2.32178500
	O	2.27318900	3.77831200	-0.35878200
	O	0.00000000	1.66146100	-3.46921300
	C	-1.41793600	3.01606600	-0.36955500
	O	-2.27318900	3.77831200	-0.35878200
	C	1.41793600	-3.01606600	-0.36955500
	C	0.00000000	-1.61899800	-2.32178500
	O	2.27318900	-3.77831200	-0.35878200
	O	0.00000000	-1.66146100	-3.46921300
	C	-1.41793600	-3.01606600	-0.36955500
	O	-2.27318900	-3.77831200	-0.35878200
	N	-1.20757500	0.00000000	-0.08254200
	N	1.20757500	0.00000000	-0.08254200
	O	-2.42860300	0.00000000	0.10280800
	O	2.42860300	0.00000000	0.10280800
	Ru	0.00000000	1.69135400	-0.41289000
	Ru	0.00000000	-1.69135400	-0.41289000
	Ru	0.00000000	0.00000000	1.66754500
	C	0.00000000	1.91339600	1.95887000
	O	0.00000000	2.83957800	2.66786100
	C	0.00000000	-1.91339600	1.95887000
	O	0.00000000	-2.83957800	2.66786100
BP86	C	1.41030900	3.07510000	-0.38456300
	C	0.00000000	1.65089900	-2.32673200
	O	2.27410900	3.85777100	-0.40045300
	O	0.00000000	1.70650800	-3.49355700
	C	-1.41030900	3.07510000	-0.38456300
	O	-2.27410900	3.85777100	-0.40045300
	C	1.41030900	-3.07510000	-0.38456300
	C	0.00000000	-1.65089900	-2.32673200
	O	2.27410900	-3.85777100	-0.40045300
	O	0.00000000	-1.70650800	-3.49355700
	C	-1.41030900	-3.07510000	-0.38456300
	O	-2.27410900	-3.85777100	-0.40045300
	N	-1.22700300	0.00000000	-0.06883800
	N	1.22700300	0.00000000	-0.06883800
	O	-2.45884600	0.00000000	0.13521500
	O	2.45884600	0.00000000	0.13521500
	Ru	0.00000000	1.74480900	-0.41307300
	Ru	0.00000000	-1.74480900	-0.41307300
	Ru	0.00000000	0.00000000	1.66872300
	C	0.00000000	1.92273100	1.98523400
	O	0.00000000	2.84073600	2.73536400
	C	0.00000000	-1.92273100	1.98523400
	O	0.00000000	-2.84073600	2.73536400

Table S13. The optimized geometry for $Ru_3(NO)_2(CO)_7$ structure **3-(NO)₂-7** (C_s).

mPW1PW91	C	-1.36223500	-0.57361800	2.68008900
	C	0.76718100	-2.35409100	2.25559600
	O	-2.27990400	-0.46506100	3.36369900
	O	1.14472600	-3.35604800	2.67634800
	C	-1.36223500	-0.57361800	-2.68008900
	C	0.76718100	-2.35409100	-2.25559600
	O	-2.27990400	-0.46506100	-3.36369900
	O	1.14472600	-3.35604800	-2.67634800
	N	1.39579000	-0.21514800	0.00000000
	N	-0.76775300	-1.44882600	0.00000000
	O	2.34991700	0.65077500	0.00000000
	O	-1.71906700	-2.16323800	0.00000000
	Ru	0.17558000	-0.69025500	1.52903900
	Ru	0.17558000	-0.69025500	-1.52903900
	Ru	0.31232100	1.56765600	0.00000000
	C	0.55826600	2.68926800	1.55640700
	O	0.67123400	3.38687100	2.46548400
	C	0.55826600	2.68926800	-1.55640700
	O	0.67123400	3.38687100	-2.46548400
	C	-1.54171600	1.73908900	0.00000000
	O	-2.69017200	1.86096100	0.00000000
BP86	C	-0.60870700	1.37744800	2.68842400
	C	-2.31591800	-0.81000400	2.31914800
	O	-0.49699400	2.28065800	3.42340500
	O	-3.28509500	-1.23913600	2.81592200
	C	-0.60870700	1.37744800	-2.68842400
	C	-2.31591800	-0.81000400	-2.31914800
	O	-0.49699400	2.28065800	-3.42340500
	O	-3.28509500	-1.23913600	-2.81592200
	N	-0.20505800	-1.41424200	0.00000000
	N	-1.53301500	0.77685200	0.00000000
	O	0.65615700	-2.39497200	0.00000000
	O	-2.30299900	1.70786500	0.00000000
	Ru	-0.70524200	-0.15212600	1.52344900
	Ru	-0.70524200	-0.15212600	-1.52344900
	Ru	1.59404700	-0.31579100	0.00000000
	C	2.70413600	-0.55731600	1.57511000
	O	3.41521200	-0.65607100	2.49939600
	C	2.70413600	-0.55731600	-1.57511000
	O	3.41521200	-0.65607100	-2.49939600
	C	1.76343300	1.54498400	0.00000000
	O	1.89998000	2.71022800	0.00000000

Table S14. The optimized geometry for $Ru_3(N)(NO)(CO)_{10}$ structure **3-NNO-10** (C_s).

mPW1PW91	N,0,-2.9459456484,1.615510545,-0.8208049504			
	N,0,-3.9808694418,0.867886807,-1.3395094154			
	Ru,0,-5.334484006,1.682125187,-2.7133752952			
	C,0,-6.3901133588,3.0332695409,-1.9790298586			
	C,0,-6.2077134295,1.8756837558,-4.3806389545			
	O,0,-7.0065962337,3.9277698974,-1.5841718801			
	O,0,-6.7380005333,1.9573752429,-5.3994925115			
	Ru,0,-5.7163968693,0.4690429062,-0.2749195493			
	Ru,0,-3.1087608529,0.2569341447,-3.2612742699			
	C,0,-2.9452688797,0.0346892408,-5.1327556867			
	C,0,-2.4851291477,2.1018338954,-3.4711544858			
	C,0,-1.4251171299,-0.5589548051,-2.7217045039			
	C,0,-4.1788770942,-1.365407627,-3.2030139029			
	C,0,-5.0461231372,1.337065754,1.3876078509			
	C,0,-6.5286777885,0.1174037704,-2.1147014629			
	C,0,-5.4401542365,-1.296845008,0.2804624152			
	C,0,-7.5002772737,0.5210569189,0.4228478581			
	O,0,-8.5660623385,0.5512717982,0.8464778148			
	O,0,-7.3323033939,-0.5052828379,-2.7102633222			
	O,0,-4.8466131785,1.7691467239,2.4294811506			
	O,0,-5.2517727417,-2.3860421565,0.597966694			
	O,0,-0.4244327026,-1.0342847798,-2.4282768396			
	O,0,-2.0839850996,3.1595167747,-3.659090358			
	O,0,-2.8613309551,-0.0963207156,-6.2719032439			
	O,0,-4.757521446,-2.3540393529,-3.202943696			
	O,0,-2.7284959817,1.498166586,0.361690209			
BP86	N	-0.25701100	1.92274000	0.00000000
	N	0.60866900	0.75546100	0.00000000
	Ru	-1.42085000	0.19310700	0.00000000
	C	-2.78437200	0.08824300	1.29281000
	C	-2.78437200	0.08824300	-1.29281000
	O	-3.62682400	0.05239800	2.10737400
	O	-3.62682400	0.05239800	-2.10737400
	Ru	0.62564700	-0.02743000	1.97160800
	Ru	0.62564700	-0.02743000	-1.97160800
	C	0.23695000	-0.64171400	-3.73306100
	C	0.89263100	1.75872900	-2.77646900
	C	2.36925400	-0.81244700	-1.90972400
	C	-0.46671000	-1.59982000	-1.33589900
	C	0.89263100	1.75872900	2.77646900
	C	-0.46671000	-1.59982000	1.33589900
	C	2.36925400	-0.81244700	1.90972400
	C	0.23695000	-0.64171400	3.73306100
	O	0.00491500	-1.00446300	4.81866600
	O	-0.84159500	-2.70907300	1.49206200
	O	1.09865200	2.74448900	3.36451600
	O	3.44285300	-1.27268800	1.90401700
	O	3.44285300	-1.27268800	-1.90401700
	O	1.09865200	2.74448900	-3.36451600
	O	0.00491500	-1.00446300	-4.81866600
	O	-0.84159500	-2.70907300	-1.49206200
	O	0.09722800	3.08539800	0.00000000

Table S15. The optimized geometry for $Ru_3(N)(NO)(CO)_9$ structure **3-NNO-9-1** (C_s).

mPW1PW91	C	-0.10292400	-1.34463600	2.89366100
	C	2.48316500	-0.66020800	2.06578600
	O	-0.57497000	-2.09108200	3.62623600
	O	3.55218700	-0.99699300	2.32301500
	C	0.63773000	1.37325100	2.86670000
	O	0.57349600	2.28403500	3.56343000
	C	-0.10292400	-1.34463600	-2.89366100
	C	2.48316500	-0.66020800	-2.06578600
	O	-0.57497000	-2.09108200	-3.62623600
	O	3.55218700	-0.99699300	-2.32301500
	C	0.63773000	1.37325100	-2.86670000
	O	0.57349600	2.28403500	-3.56343000
	N	1.34621100	0.99397800	0.00000000
	N	0.07882600	-1.06464100	0.00000000
	O	1.94851000	2.03468900	0.00000000
	Ru	0.70563000	-0.12116100	1.63737800
	Ru	0.70563000	-0.12116100	-1.63737800
	Ru	-1.54807600	-0.14611400	0.00000000
	C	-2.75515500	-0.36705800	1.49251100
	C	-2.75515500	-0.36705800	-1.49251100
	C	-1.68075600	1.74088000	0.00000000
	O	-3.48685800	-0.46436200	2.37487400
	O	-1.70429200	2.89266100	0.00000000
	O	-3.48685800	-0.46436200	-2.37487400
BP86	C	-0.09161300	-1.33276700	2.94016000
	C	2.50401600	-0.67822400	2.08549900
	O	-0.56210600	-2.07898600	3.70368100
	O	3.58692400	-1.02336900	2.36061000
	C	0.66232600	1.37888200	2.88806100
	O	0.60212000	2.30632400	3.59508500
	C	-0.09161300	-1.33276700	-2.94016000
	C	2.50401600	-0.67822400	-2.08549900
	O	-0.56210600	-2.07898600	-3.70368100
	O	3.58692400	-1.02336900	-2.36061000
	C	0.66232600	1.37888200	-2.88806100
	O	0.60212000	2.30632400	-3.59508500
	N	1.36666800	0.99987100	0.00000000
	N	0.06517900	-1.05048700	0.00000000
	O	1.98376100	2.05221700	0.00000000
	Ru	0.72119100	-0.12344600	1.66195300
	Ru	0.72119100	-0.12344600	-1.66195300
	Ru	-1.57716300	-0.12810100	0.00000000
	C	-2.78589700	-0.40157900	1.48780800
	C	-2.78589700	-0.40157900	-1.48780800
	C	-1.74795300	1.76986800	0.00000000
	O	-3.54041800	-0.53511300	2.37150100
	O	-1.79065200	2.93995500	0.00000000
	O	-3.54041800	-0.53511300	-2.37150100

Table S16. The optimized geometry for $Ru_3(N)(NO)(CO)_9$ structure **3-NNO-9-2** (C_s).

mPW1PW91	C	-0.57382200	1.48408700	2.85005000
	C	-2.58152500	-0.12125400	1.83316900
	O	-0.47109100	2.38940000	3.54872500
	O	-3.72780700	-0.18157300	1.90853600
	C	-0.30316500	-1.29149300	3.05777000
	O	-0.04516300	-2.08520400	3.84694100
	C	-0.57382200	1.48408700	-2.85005000
	C	-2.58152500	-0.12125400	-1.83316900
	O	-0.47109100	2.38940000	-3.54872500
	O	-3.72780700	-0.18157300	-1.90853600
	C	-0.30316500	-1.29149300	-3.05777000
	O	-0.04516300	-2.08520400	-3.84694100
	N	-0.34111200	-1.20855400	0.00000000
	N	-0.54980200	1.13733700	0.00000000
	O	0.54351900	-2.12589400	0.00000000
	Ru	-0.70363800	-0.00349800	1.65354600
	Ru	-0.70363800	-0.00349800	-1.65354600
	Ru	1.43469900	-0.05655800	0.00000000
	C	2.52420400	-0.44505100	1.54914300
	O	3.20265700	-0.67721000	2.44678500
	C	2.52420400	-0.44505100	-1.54914300
	O	3.20265700	-0.67721000	-2.44678500
	C	1.88208500	1.76787600	0.00000000
	O	2.15791400	2.88158400	0.00000000
BP86	C	-0.58345300	1.47275900	2.91648100
	C	-2.60051200	-0.10055300	1.84399300
	O	-0.47656100	2.37044900	3.65634000
	O	-3.76574500	-0.15762100	1.93029100
	C	-0.33837500	-1.29184200	3.08806100
	O	-0.09831500	-2.12718800	3.86870100
	C	-0.58345300	1.47275900	-2.91648100
	C	-2.60051200	-0.10055300	-1.84399300
	O	-0.47656100	2.37044900	-3.65634000
	O	-3.76574500	-0.15762100	-1.93029100
	C	-0.33837500	-1.29184200	-3.08806100
	O	-0.09831500	-2.12718800	-3.86870100
	N	-0.34654700	-1.20931000	0.00000000
	N	-0.54491400	1.14135900	0.00000000
	O	0.55198900	-2.14676500	0.00000000
	Ru	-0.71368200	0.00917500	1.68615500
	Ru	-0.71368200	0.00917500	-1.68615500
	Ru	1.45873600	-0.06012400	0.00000000
	C	2.55107400	-0.45406100	1.55321800
	O	3.24744900	-0.69103500	2.45981900
	C	2.55107400	-0.45406100	-1.55321800
	O	3.24744900	-0.69103500	-2.45981900
	C	1.94178500	1.76048500	0.00000000
	O	2.24239300	2.88695600	0.00000000

Table S17. The optimized geometry for $Ru_3(N)(NO)(CO)_8$ structure **3-NNO-8** (C_s).

mPW1PW91	C	0.10282400	-1.42063300	2.94637300
	C	2.33331700	-0.23335500	1.77875200
	O	-0.10382000	-2.26806000	3.69102400
	O	3.46743400	-0.36860400	1.90252600
	C	0.29698200	1.37737600	2.98516000
	O	0.23059700	2.24305000	3.73376200
	C	0.10282400	-1.42063300	-2.94637300
	C	2.33331700	-0.23335500	-1.77875200
	O	-0.10382000	-2.26806000	-3.69102400
	O	3.46743400	-0.36860400	-1.90252600
	C	0.29698200	1.37737600	-2.98516000
	O	0.23059700	2.24305000	-3.73376200
	N	0.63481600	1.24689600	0.00000000
	N	0.09981100	-1.17455400	0.00000000
	O	0.85908600	2.42652800	0.00000000
	Ru	0.44284200	-0.02687700	1.65791600
	Ru	0.44284200	-0.02687700	-1.65791600
	Ru	-1.64986600	-0.70328000	0.00000000
	C	-2.09774300	0.39726200	1.46495700
	O	-2.72018800	1.14006100	2.11993400
	C	-2.09774300	0.39726200	-1.46495700
	O	-2.72018800	1.14006100	-2.11993400
BP86	C	0.12151900	-1.42223400	2.98911200
	C	2.36072600	-0.22508400	1.81112100
	O	-0.08433400	-2.27483400	3.75833200
	O	3.51173000	-0.35498500	1.96566400
	C	0.30497300	1.37096000	3.01297800
	O	0.23874300	2.25584800	3.76934800
	C	0.12151900	-1.42223400	-2.98911200
	C	2.36072600	-0.22508400	-1.81112100
	O	-0.08433400	-2.27483400	-3.75833200
	O	3.51173000	-0.35498500	-1.96566400
	C	0.30497300	1.37096000	-3.01297800
	O	0.23874300	2.25584800	-3.76934800
	N	0.63438700	1.26048000	0.00000000
	N	0.09640900	-1.15502200	0.00000000
	O	0.85400200	2.45866500	0.00000000
	Ru	0.46264100	-0.03476400	1.68571800
	Ru	0.46264100	-0.03476400	-1.68571800
	Ru	-1.67552400	-0.68091800	0.00000000
	C	-2.18175100	0.36564700	1.50236400
	O	-2.80380200	1.09526200	2.20103500
	C	-2.18175100	0.36564700	-1.50236400
	O	-2.80380200	1.09526200	-2.20103500

Table S18. The optimized geometry for $Ru_3(N)_2(CO)_{10}$ structure **3-NN-10** (C_s).

mPW1PW91	N	1.65288000	2.83826100	0.00000000
	N	1.41817700	1.74617100	0.00000000
	Ru	1.15265200	-0.29723800	0.00000000
	C	2.43679000	-0.88883000	1.25826700
	C	2.43679000	-0.88883000	-1.25826700
	O	3.22964200	-1.29127800	1.99153900
	O	3.22964200	-1.29127800	-1.99153900
	Ru	-0.66984400	0.36391400	2.17086800
	Ru	-0.66984400	0.36391400	-2.17086800
	C	-0.10644400	-1.37941300	-1.37036300
	C	-2.49820400	0.07111200	-1.62468300
	C	-1.29468400	0.10709600	-3.91569400
	C	0.99476300	1.06389700	-2.92682900
	C	-2.49820400	0.07111200	1.62468300
	C	0.99476300	1.06389700	2.92682900
	C	-1.29468400	0.10709600	3.91569400
	C	-0.10644400	-1.37941300	1.37036300
	O	-0.36169400	-2.50318300	1.58116100
	O	1.90381000	1.57026600	3.40958000
	O	-3.62658100	-0.03098600	1.43999800
	O	-1.62357900	-0.16502700	4.98792000
	O	-1.62357900	-0.16502700	-4.98792000
	O	-3.62658100	-0.03098600	-1.43999800
	O	-0.36169400	-2.50318300	-1.58116100
	O	1.90381000	1.57026600	-3.40958000
BP86	N	1.41276700	2.89200900	0.00000000
	N	1.25438000	1.76368300	0.00000000
	Ru	1.16313700	-0.27091000	0.00000000
	C	2.49438000	-0.77916900	1.26321200
	C	2.49438000	-0.77916900	-1.26321200
	O	3.33917500	-1.13458100	1.99253600
	O	3.33917500	-1.13458100	-1.99253600
	Ru	-0.66281200	0.31596900	2.24132000
	Ru	-0.66281200	0.31596900	-2.24132000
	C	-0.06662300	-1.40351800	-1.38909600
	C	-2.49586500	0.13567300	-1.64973200
	C	-1.30639600	-0.03135700	-3.96977200
	C	0.96714000	1.06588600	-3.02722000
	C	-2.49586500	0.13567300	1.64973200
	C	0.96714000	1.06588600	3.02722000
	C	-1.30639600	-0.03135700	3.96977200
	C	-0.06662300	-1.40351800	1.38909600
	O	-0.29688400	-2.55474100	1.58173100
	O	1.84359200	1.64365300	3.53587900
	O	-3.64600900	0.13615900	1.44771200
	O	-1.65439200	-0.36081500	5.04020600
	O	-1.65439200	-0.36081500	-5.04020600
	O	-3.64600900	0.13615900	-1.44771200
	O	-0.29688400	-2.55474100	-1.58173100
	O	1.84359200	1.64365300	-3.53587900

Table S19. The optimized geometry for $Ru_3(N)_2(CO)_9$ structure **3-NN-9** (C_s).

mPW1PW91	C,0,-0.1091470561,-1.3448561919,3.2401807131			
	C,0,2.4406090037,-0.7225320666,2.2979402386			
	O,0,-0.5694474506,-2.0642893203,4.0085686474			
	O,0,3.5258866812,-1.0165327652,2.5354342297			
	C,0,0.6747001477,1.2756655086,3.0762956728			
	O,0,0.6799903378,2.2123483236,3.7427397845			
	C,0,-0.1091470561,-1.3448561919,-3.2401807131			
	C,0,2.4406090037,-0.7225320666,-2.2979402386			
	O,0,-0.5694474506,-2.0642893203,-4.0085686474			
	O,0,3.5258866812,-1.0165327652,-2.5354342297			
	C,0,0.6747001477,1.2756655086,-3.0762956728			
	O,0,0.6799903378,2.2123483236,-3.7427397845			
	N,0,0.6939388148,0.3982071273,0.			
	N,0,0.2680746109,-0.9533007527,0.			
	Ru,0,0.658443538,-0.2141458933,1.9052813886			
	Ru,0,0.658443538,-0.2141458933,-1.9052813886			
	Ru,0,-1.3343502949,0.2998203125,0.			
	C,0,-2.5121006536,-0.2852274213,1.3759077339			
	C,0,-2.5121006536,-0.2852274213,-1.3759077339			
	C,0,-1.8952659241,2.1503231745,0.			
	O,0,-3.269375684,-0.6356563623,2.1728715401			
	O,0,-2.168276739,3.268592815,0.			
	O,0,-3.269375684,-0.6356563623,-2.1728715401			
BP86	C	0.27912300	-1.32156000	3.24973000
	C	2.59992500	-0.07337000	2.31929900
	O	0.01229200	-2.15871800	4.01990700
	O	3.74137200	-0.09062000	2.57045400
	C	0.38846200	1.42020900	3.11620300
	O	0.15809400	2.33810000	3.80270100
	C	0.27912300	-1.32156000	-3.24973000
	C	2.59992500	-0.07337000	-2.31929900
	O	0.01229200	-2.15871800	-4.01990700
	O	3.74137200	-0.09062000	-2.57045400
	C	0.38846200	1.42020900	-3.11620300
	O	0.15809400	2.33810000	-3.80270100
	N	0.60402200	0.61059600	0.00000000
	N	0.51237700	-0.83280100	0.00000000
	Ru	0.74176700	-0.01868200	1.92719200
	Ru	0.74176700	-0.01868200	-1.92719200
	Ru	-1.36818700	0.01832100	0.00000000
	C	-2.38961600	-0.82549900	1.37812700
	C	-2.38961600	-0.82549900	-1.37812700
	C	-2.34183800	1.69265600	0.00000000
	O	-3.06170300	-1.34162800	2.18789100
	O	-2.87183200	2.73574000	0.00000000
	O	-3.06170300	-1.34162800	-2.18789100

Table S20. The optimized geometry for $Ru_3(N)_2(CO)_8$ structure **3-NN-8** (C_s).

mPW1PW91	C	-0.20070100	-1.15422100	3.24304900
	C	2.38481500	-0.48180000	2.38392300
	O	-0.67370000	-1.86970900	4.00775000
	O	3.46841700	-0.73749700	2.66828100
	C	0.51444400	1.46845000	3.08149300
	O	0.46086100	2.40773100	3.74362400
	C	-0.20070100	-1.15422100	-3.24304900
	C	2.38481500	-0.48180000	-2.38392300
	O	-0.67370000	-1.86970900	-4.00775000
	O	3.46841700	-0.73749700	-2.66828100
	C	0.51444400	1.46845000	-3.08149300
	O	0.46086100	2.40773100	-3.74362400
	N	0.66100800	0.56527900	0.00000000
	N	0.26252400	-0.75839500	0.00000000
	Ru	0.59289600	-0.02612700	1.92794000
	Ru	0.59289600	-0.02612700	-1.92794000
	Ru	-1.35616900	0.41292300	0.00000000
	C	-2.55034700	-0.11280100	1.36338600
	C	-2.55034700	-0.11280100	-1.36338600
	O	-3.30224600	-0.49759500	2.15479100
	O	-3.30224600	-0.49759500	-2.15479100
BP86	C	0.17003200	1.17832800	3.26628100
	C	-2.41192700	0.44771200	2.42521800
	O	0.63526300	1.92167100	4.03850800
	O	-3.51587900	0.67964100	2.73060800
	C	-0.48794800	-1.46682400	3.11383800
	O	-0.40787200	-2.41929100	3.78846800
	C	0.17003200	1.17832800	-3.26628100
	C	-2.41192700	0.44771200	-2.42521800
	O	0.63526300	1.92167100	-4.03850800
	O	-3.51587900	0.67964100	-2.73060800
	C	-0.48794800	-1.46682400	-3.11383800
	O	-0.40787200	-2.41929100	-3.78846800
	N	-0.66495100	-0.57619800	0.00000000
	N	-0.27431600	0.77042200	0.00000000
	Ru	-0.60847000	0.02787800	1.95689700
	Ru	-0.60847000	0.02787800	-1.95689700
	Ru	1.38091600	-0.40470800	0.00000000
	C	2.58675300	0.11048600	1.36304500
	C	2.58675300	0.11048600	-1.36304500
	O	3.35579800	0.49034500	2.16839200
	O	3.35579800	0.49034500	-2.16839200

Table S21. The optimized geometry for $Ru_3(N)(NCO)(CO)_9$ structure **3-NNCO-9-1** (C_s).

mPW1PW91	C	-0.33585400	-1.38483700	2.80913300
	C	2.29822700	-1.03194200	2.12607000
	O	-0.90934800	-2.08421300	3.51885000
	O	3.30029800	-1.51300900	2.41702900
	C	0.64975400	1.20152200	2.90277500
	O	0.62867700	2.09580700	3.62264700
	C	-0.33585400	-1.38483700	-2.80913300
	C	2.29822700	-1.03194200	-2.12607000
	O	-0.90934800	-2.08421300	-3.51885000
	O	3.30029800	-1.51300900	-2.41702900
	C	0.64975400	1.20152200	-2.90277500
	O	0.62867700	2.09580700	-3.62264700
	N	1.53722900	0.84946600	0.00000000
	N	-0.09338100	-1.11413900	0.00000000
	Ru	0.61508200	-0.27233800	1.63719900
	Ru	0.61508200	-0.27233800	-1.63719900
	Ru	-1.62593200	-0.01638500	0.00000000
	C	-2.87439500	-0.05020800	1.46717600
	C	-2.87439500	-0.05020800	-1.46717600
	C	-1.48587600	1.86096500	0.00000000
	O	-3.62175900	-0.03539200	2.34192500
	O	-1.33594500	3.00331300	0.00000000
	O	-3.62175900	-0.03539200	-2.34192500
	C	2.37423900	1.73267600	0.00000000
	O	3.18069900	2.59069300	0.00000000
BP86	C	-0.32180300	-1.36428400	2.85502600
	C	2.31497100	-1.03750600	2.17418400
	O	-0.89928200	-2.05941000	3.59663000
	O	3.32508300	-1.52471600	2.50301300
	C	0.65879000	1.22303100	2.90250300
	O	0.62750400	2.14516700	3.61794700
	C	-0.32180300	-1.36428400	-2.85502600
	C	2.31497100	-1.03750600	-2.17418400
	O	-0.89928200	-2.05941000	-3.59663000
	O	3.32508300	-1.52471600	-2.50301300
	C	0.65879000	1.22303100	-2.90250300
	O	0.62750400	2.14516700	-3.61794700
	N	1.56317700	0.84308700	0.00000000
	N	-0.09579900	-1.09941400	0.00000000
	Ru	0.63471900	-0.27286400	1.66060000
	Ru	0.63471900	-0.27286400	-1.66060000
	Ru	-1.65154900	-0.00250500	0.00000000
	C	-2.91469600	-0.09804700	1.45466400
	C	-2.91469600	-0.09804700	-1.45466400
	C	-1.58296900	1.89208600	0.00000000
	O	-3.69261700	-0.12505000	2.32819000
	O	-1.46620900	3.05738500	0.00000000
	O	-3.69261700	-0.12505000	-2.32819000
	C	2.45295400	1.69747800	0.00000000
	O	3.30410800	2.53323500	0.00000000

Table S22. The optimized geometry for $Ru_3(N)(NCO)(CO)_9$ structure **3-NNCO-9-2** (C_s).

mPW1PW91	C	-0.00000400	-1.43418100	1.51436000
	C	-1.39804100	-2.74593000	-0.79122100
	O	0.00000200	-2.07091700	2.52108800
	O	-2.23844900	-3.52017400	-0.94791300
	C	0.00001300	2.47580900	-1.63614900
	O	0.00002000	3.31853300	-2.45492700
	C	-2.90350400	-0.48142400	1.09098800
	O	-3.66966000	-1.13740700	1.63971400
	C	-2.83836000	0.73460400	-1.31304700
	O	-3.51747800	0.77831900	-2.23912400
	Ru	-0.00001100	-1.50844800	-0.45886500
	Ru	-1.67062700	0.61890000	0.19054700
	Ru	1.67062100	0.61889000	0.19055300
	C	2.83837200	0.73461100	-1.31302900
	C	2.27104200	2.22104200	1.06318800
	O	2.64454600	3.16499000	1.59898700
	O	3.51750200	0.77833900	-2.23909600
	N	0.00000300	1.59737200	-0.78900000
	O	3.66964800	-1.13740400	1.63974500
	C	1.39807900	-2.74585300	-0.79124400
	C	-2.27106200	2.22105700	1.06316800
	O	2.23853100	-3.52004300	-0.94796100
	O	-2.64458700	3.16500400	1.59895500
	C	2.90349500	-0.48142700	1.09100700
	N	-0.00000600	-0.11663400	1.10297400
BP86	C	1.27632900	-1.69590700	0.00000000
	C	2.85373900	0.50628000	-1.39480800
	O	1.79219900	-2.78617300	0.00000000
	O	3.65329900	0.60955000	-2.24682400
	C	-2.32999200	1.88388100	0.00000000
	O	-3.10798600	2.78490300	0.00000000
	C	0.34240200	-1.13140300	-2.93743400
	O	0.93688300	-1.75423700	-3.72647600
	C	-0.58889100	1.39738900	-2.84634400
	O	-0.52222500	2.34373800	-3.52886300
	Ru	1.58712800	0.27432200	0.00000000
	Ru	-0.65039500	-0.12360400	-1.69186500
	Ru	-0.65039500	-0.12360400	1.69186500
	C	-0.58889100	1.39738900	2.84634400
	C	-2.33667200	-0.81022300	2.31185200
	O	-3.34239700	-1.24126200	2.71973700
	O	-0.52222500	2.34373800	3.52886300
	N	-1.51491000	0.95332100	0.00000000
	O	0.93688300	-1.75423700	3.72647600
	C	2.85373900	0.50628000	1.39480800
	C	-2.33667200	-0.81022300	-2.31185200
	O	3.65329900	0.60955000	2.24682400
	O	-3.34239700	-1.24126200	-2.71973700
	C	0.34240200	-1.13140300	2.93743400
	N	0.00000000	-1.12182100	0.00000000

Table S23. The optimized geometry for $Ru_3(N)(NCO)(CO)_8$ structure **3-NNCO-8** (C_s).

mPW1PW91	C	-1.91226965	-0.58244185	2.86006336
	C	0.60503450	-0.65391027	1.77093000
	O	-2.51082312	-1.24229495	3.58575713
	O	1.51365269	-1.34439300	1.89148824
	C	-0.41517740	1.72182440	3.04609429
	O	-0.08113138	2.47932140	3.83824572
	C	-1.91226965	-0.58244185	-2.86006336
	C	0.60503450	-0.65391027	-1.77093000
	O	-2.51082312	-1.24229495	-3.58575713
	O	1.51365269	-1.34439300	-1.89148824
	C	-0.41517740	1.72182440	-3.04609429
	O	-0.08113138	2.47932140	-3.83824572
	N	-0.03305359	1.60203906	0.00000000
	N	-1.76447981	-0.36808730	0.00000000
	Ru	-0.93789700	0.46753143	1.65261117
	Ru	-0.93789700	0.46753143	-1.65261117
	Ru	-3.10346224	0.86948326	0.00000000
	C	-2.92683450	2.07134596	1.43157578
	O	-3.08124259	3.04643483	2.06044328
	C	-2.92683450	2.07134596	-1.43157578
	O	-3.08124259	3.04643483	-2.06044328
	C	0.74590096	2.53715284	0.00000000
	O	1.50116368	3.44075641	0.00000000
BP86	C	-0.19981800	-1.47751100	2.90194800
	C	2.22576000	-0.75409300	1.81687200
	O	-0.56195500	-2.29246300	3.65759000
	O	3.31857000	-1.12719900	1.98889300
	C	0.48282600	1.17943400	3.05624800
	O	0.56140000	2.03210400	3.84680200
	C	-0.19981800	-1.47751100	-2.90194800
	C	2.22576000	-0.75409300	-1.81687200
	O	-0.56195500	-2.29246300	-3.65759000
	O	3.31857000	-1.12719900	-1.98889300
	C	0.48282600	1.17943400	-3.05624800
	O	0.56140000	2.03210400	-3.84680200
	N	0.87973900	1.18947700	0.00000000
	N	-0.12292800	-1.23428000	0.00000000
	Ru	0.40050600	-0.18962400	1.67450400
	Ru	0.40050600	-0.18962400	-1.67450400
	Ru	-1.81902400	-0.50251500	0.00000000
	C	-2.10359900	0.66665700	1.45506300
	O	-2.56960400	1.53396900	2.11900300
	C	-2.10359900	0.66665700	-1.45506300
	O	-2.56960400	1.53396900	-2.11900300
	C	1.35738100	2.32658700	0.00000000
	O	1.81424300	3.42940000	0.00000000

Table S24. The optimized geometry for $Ru_3(N)(NCO)(CO)_7$ structure **3-NNCO-7** (C_1).

mPW1PW91	C	-0.19680400	-2.89623400	-0.76767700
	O	-0.27495800	-3.94436000	-1.22881300
	C	-0.08038800	-1.88782300	1.59883100
	O	-0.08732800	-2.28616200	2.68200500
	C	2.86882500	2.10716400	-0.81148200
	O	3.55621400	2.73936500	-1.48920700
	C	3.00576500	1.02218100	1.60130500
	O	3.70683000	0.99722000	2.51458000
	N	2.05096700	-0.79278300	-0.54598200
	N	-0.05220100	0.89054400	-0.41151800
	Ru	-0.06307200	-1.13510000	-0.07730200
	Ru	1.71211900	1.06341600	0.18212700
	Ru	-1.94504000	0.80533800	-0.03089100
	C	-2.24226900	-1.09394600	-0.27388500
	C	-2.95903700	1.73405900	-1.27278600
	C	-3.40572200	0.98332300	1.15208300
	O	-3.03351200	-1.93753200	-0.48439600
	O	-4.24224800	1.14407000	1.93054200
	O	-3.59110100	2.24375800	-2.09511100
	C	2.98780500	-1.39527400	-1.04544700
	O	3.86151400	-2.00708600	-1.53412500
BP86	C	-0.10159800	-2.97495800	-0.67033800
	O	-0.12573100	-4.07159600	-1.06958100
	C	-0.02031400	-1.82462000	1.64816900
	O	0.00046500	-2.18460600	2.76511100
	C	2.78303500	2.27864600	-0.71704700
	O	3.42015800	3.04393400	-1.34195500
	C	2.99384500	0.98524700	1.59685200
	O	3.67132100	0.94915900	2.55388000
	N	2.03551800	-0.78090000	-0.70225100
	N	-0.07711700	0.93792700	-0.35368900
	Ru	-0.05373900	-1.17228400	-0.07182200
	Ru	1.71441100	1.05612900	0.16871000
	Ru	-1.96698900	0.81092500	-0.03949300
	C	-2.19447000	-1.14699300	-0.21658600
	C	-2.99466200	1.57269400	-1.38739400
	C	-3.43838500	1.06264400	1.14986500
	O	-3.02592000	-1.98910700	-0.37640600
	O	-4.27826300	1.27862800	1.93862000
	O	-3.62530400	2.00098900	-2.28135100
	C	3.00545400	-1.35515600	-1.21018200
	O	3.90973900	-1.93415900	-1.72504800

Table S25. The optimized geometry for $Ru_3(NO)(NCO)(CO)_{10}$ structure **3-NONCO-10** (C_s).

mPW1PW91	C	-1.79194317	1.82478754	0.00025486
	C	-2.83741014	-0.30465310	-1.48505420
	O	-1.90274463	2.96794920	0.00028186
	O	-3.59029711	-0.43767503	-2.34547101
	C	-1.17489677	-1.97892894	0.00012559
	O	-0.93669242	-3.10347789	0.00007860
	C	-0.17030015	1.38265985	-2.62863881
	O	1.19298678	2.61776269	-0.00012415
	O	-0.70569633	2.15166077	-3.29501140
	C	0.21325287	-1.33252826	-2.70202264
	O	-0.09885719	-2.20588854	-3.37691099
	Ru	-1.65065223	-0.10788090	0.00021551
	Ru	0.74368541	0.14332914	-1.55552771
	Ru	0.74409261	0.14329354	1.55533999
	C	0.21395775	-1.33258935	2.70194169
	C	2.44392587	0.44515432	2.39127435
	O	3.43739575	0.65928680	2.92383478
	O	-0.09798225	-2.20596339	3.37689071
	N	1.58347777	-1.11553556	-0.00021700
	O	-0.70482005	2.15158893	3.29525636
	C	-2.83699045	-0.30468847	1.48581561
	C	2.44329850	0.44521415	-2.39190090
	O	-3.58963139	-0.43773066	2.34644463
	O	3.43662899	0.65935571	-2.92471787
	C	-0.16960522	1.38260235	2.62872174
	N	1.00709437	1.42735782	-0.00011332
	C	2.31818444	-2.08121786	-0.00033173
	O	3.03116015	-3.01948508	-0.00044324
BP86	C	-1.13011500	-2.32997500	0.00000000
	C	1.24010300	-2.61196900	-1.48861200
	O	-2.18772900	-2.82328900	0.00000000
	O	1.62994200	-3.30148400	-2.34930100
	C	2.27033200	-0.47785800	0.00000000
	O	3.27017500	0.12552500	0.00000000
	C	-1.25559500	-0.60661200	-2.65965200
	O	-2.91405700	0.22662600	0.00000000
	O	-1.80299200	-1.37301300	-3.35195300
	C	1.17595900	0.67901700	-2.71734700
	O	2.11262600	0.68592900	-3.41187700
	Ru	0.65547600	-1.55356100	0.00000000
	Ru	-0.40156300	0.67462000	-1.57338900
	Ru	-0.40156300	0.67462000	1.57338900
	C	1.17595900	0.67901700	2.71734700
	C	-1.25559500	2.16493900	2.42800800
	O	-1.79755400	3.02976600	2.99442200
	O	2.11262600	0.68592900	3.41187700
	N	0.49813300	1.89415300	0.00000000
	O	-1.80299200	-1.37301300	3.35195300
	C	1.24010300	-2.61196900	1.48861200
	C	-1.25559500	2.16493900	-2.42800800
	O	1.62994200	-3.30148400	2.34930100
	O	-1.79755400	3.02976600	-2.99442200
	C	-1.25559500	-0.60661200	2.65965200

	N	-1.71750800	0.47532900	0.00000000
	C	1.17585800	2.91965500	0.00000000
	O	1.83224300	3.91748100	0.00000000

Table S26. The optimized geometry for $Ru_3(NO)(NCO)(CO)_9$ structure **3-NONCO-9** (C_s).

mPW1PW91	C	2.60584900	0.17562300	0.00000000
	C	0.07962000	2.64778500	-1.44162000
	O	3.75834700	0.35494400	0.00000000
	O	0.23777000	3.43273400	-2.26947700
	C	-2.06446100	1.72604800	0.00000000
	O	-3.20638400	1.88492900	0.00000000
	C	1.05159600	0.19578900	-3.05477000
	O	1.61966000	0.73342000	-3.89596700
	C	-1.49690300	-0.63574700	-2.65485900
	O	-2.46894300	-0.59488400	-3.26840500
	Ru	-0.22492200	1.43792700	0.00000000
	Ru	0.07963200	-0.72510400	-1.65126500
	Ru	0.07963200	-0.72510400	1.65126500
	C	-1.49690300	-0.63574700	2.65485900
	C	0.63052500	-2.48690500	2.20868400
	O	0.88176000	-3.55148900	2.55978500
	O	-2.46894300	-0.59488400	3.26840500
	N	-0.92040400	-1.36126700	0.00000000
	O	1.61966000	0.73342000	3.89596700
	O	-1.94613900	-2.00785500	0.00000000
	C	0.07962000	2.64778500	1.44162000
	C	0.63052500	-2.48690500	-2.20868400
	O	0.23777000	3.43273400	2.26947700
	O	0.88176000	-3.55148900	-2.55978500
	C	1.05159600	0.19578900	3.05477000
	N	1.39068200	-0.02362000	0.00000000
BP86	C	2.06068100	1.60425800	0.00000000
	C	-1.41289600	2.27504100	-1.44386200
	O	2.93129200	2.40497200	0.00000000
	O	-1.71832800	3.03820400	-2.27784600
	C	-2.71185400	0.32103700	0.00000000
	O	-3.77522900	-0.17188700	0.00000000
	C	0.81150500	0.73018600	-3.08349500
	O	1.00877700	1.49290500	-3.94576500
	C	-0.88414000	-1.35735600	-2.70831600
	O	-1.71835100	-1.86180700	-3.35415000
	Ru	-1.00627500	1.09077900	0.00000000
	Ru	0.47569800	-0.57084500	-1.67801200
	Ru	0.47569800	-0.57084500	1.67801200
	C	-0.88414000	-1.35735600	2.70831600
	C	1.90671200	-1.74310200	2.22271300
	O	2.71481100	-2.50900000	2.57754700
	O	-1.71835100	-1.86180700	3.35415000
	N	-0.03523300	-1.66082600	0.00000000
	O	1.00877700	1.49290500	3.94576500
	O	-0.54772200	-2.77774600	0.00000000
	C	-1.41289600	2.27504100	1.44386200
	C	1.90671200	-1.74310200	-2.22271300
	O	-1.71832800	3.03820400	2.27784600

	O	2.71481100	-2.50900000	-2.57754700
	C	0.81150500	0.73018600	3.08349500
	N	1.15114200	0.75006500	0.00000000

Table S27. The optimized geometry for $Ru_4(N)(NO)(CO)_{12}$ structure **4-NNO-12** (C_{2v}).

mPW1PW91	C	2.46400177	-1.44536142	1.56343905
	O	2.97812652	-2.28035632	2.16391010
	C	3.00379684	0.00016376	-0.74842912
	O	3.81789831	0.00012914	-1.55704686
	C	0.00031038	-3.42714953	0.47799518
	O	0.00039647	-4.29945092	1.22710969
	C	1.37467728	-2.57673730	-1.80224107
	O	2.19470490	-2.97877815	-2.50267734
	Ru	1.64207186	0.00022109	0.62853598
	Ru	0.00015974	-1.89810870	-0.69404434
	Ru	-0.00017486	1.89793156	-0.69452336
	C	1.37422538	2.57651827	-1.80289197
	C	-1.37471893	2.57627326	-1.80286366
	O	-2.19476643	2.97813682	-2.50337827
	O	2.19418024	2.97852587	-2.50343246
	N	-0.00000816	-0.00010026	-0.79194313
	O	-0.00036956	4.29979084	1.22598568
	C	2.46374315	1.44619265	1.56306681
	C	-1.37426603	-2.57698315	-1.80220577
	O	2.97772000	2.28143287	2.16332317
	O	-2.19423959	-2.97916731	-2.50262299
	C	-0.00029611	3.42728332	0.47711134
	Ru	-1.64205868	-0.00006732	0.62857045
	C	-3.00381248	-0.00036316	-0.74836730
	C	-2.46371178	-1.44579833	1.56348759
	C	-2.46396765	1.44575478	1.56312270
	O	-3.81793066	-0.00053722	-1.55696828
	O	-2.97766500	-2.28089168	2.16396859
	O	-2.97807774	2.28090712	2.16338733
	N	0.00001899	0.00022658	1.84798655
	O	0.00003182	0.00036285	3.05363608
BP86	C	1.44307200	2.50527500	1.57538000
	O	2.28177400	3.04171800	2.18705700
	C	0.00000000	3.04272200	-0.74061500
	O	0.00000000	3.88714600	-1.54521100
	C	3.44841800	0.00000000	0.46933500
	O	4.32988300	0.00000000	1.23733700
	C	2.60192600	1.37931400	-1.82422400
	O	3.01746600	2.20699600	-2.53965900
	Ru	0.00000000	1.66925900	0.63511300
	Ru	1.91974300	0.00000000	-0.71155200
	Ru	-1.91974300	0.00000000	-0.71155200
	C	-2.60192600	1.37931400	-1.82422400
	C	-2.60192600	-1.37931400	-1.82422400
	O	-3.01746600	-2.20699600	-2.53965900
	O	-3.01746600	2.20699600	-2.53965900
	N	0.00000000	0.00000000	-0.79565300
	O	-4.32988300	0.00000000	1.23733700
	C	-1.44307200	2.50527500	1.57538000

	C	2.60192600	-1.37931400	-1.82422400
	O	-2.28177400	3.04171800	2.18705700
	O	3.01746600	-2.20699600	-2.53965900
	C	-3.44841800	0.00000000	0.46933500
	Ru	0.00000000	-1.66925900	0.63511300
	C	0.00000000	-3.04272200	-0.74061500
	C	1.44307200	-2.50527500	1.57538000
	C	-1.44307200	-2.50527500	1.57538000
	O	0.00000000	-3.88714600	-1.54521100
	O	2.28177400	-3.04171800	2.18705700
	O	-2.28177400	-3.04171800	2.18705700
	N	0.00000000	0.00000000	1.86322600
	O	0.00000000	0.00000000	3.08631400

Table S28. The optimized geometry for $Ru_4(N)(NO)(CO)_{11}$ structure **4-NNO-11**(C_s).

mPW1PW91	C	-1.02271041	-2.78500298	-1.39684082
	O	-1.39837633	-3.51526332	-2.20508314
	C	-0.32497934	-0.18885636	-3.47404988
	O	-1.03666701	-0.30986887	-4.36957467
	C	2.03320710	-1.28227795	-2.48312454
	O	2.82467181	-2.05228940	-2.80894536
	Ru	-0.43440217	-1.63476105	0.00070963
	Ru	0.77753209	0.00073933	-1.90976561
	Ru	0.77758309	0.00232017	1.90979212
	C	2.03328470	-1.28022630	2.48413530
	C	1.74811741	1.48705241	2.59038927
	O	2.36740527	2.37029385	2.99143786
	O	2.82476073	-2.04997737	2.81054365
	N	0.86218739	0.02158596	0.00000399
	O	-1.03652350	-0.30634030	4.36991529
	C	-1.02271363	-2.78387177	1.39918889
	C	1.74805933	1.48491202	-2.59158388
	O	-1.39838832	-3.51347547	2.20801963
	O	2.36734778	2.36782708	-2.99334978
	C	-0.32487360	-0.18603021	3.47426612
	Ru	-0.85690964	1.35990237	-0.00053127
	C	0.27710300	2.88725060	-0.00118482
	C	-1.87977117	2.01702134	-1.47677046
	C	-1.87968225	2.01820786	1.47524122
	O	0.94971160	3.81763650	-0.00157759
	O	-2.52437478	2.45142594	-2.32392944
	O	-2.52423569	2.45330084	2.32208538
	N	-1.91563110	-0.47616835	0.00023927
	O	-3.08263985	-0.71001421	0.00034364
BP86	C	1.38100900	-2.82337900	-0.95784400
	O	2.17612200	-3.63922800	-1.23238000
	C	3.51313800	-0.18649300	-0.28697200
	O	4.42709800	-0.30506700	-1.00672900
	C	2.49223500	-1.30214600	2.06293700
	O	2.81953100	-2.08490400	2.86905000
	Ru	-0.00000100	-1.58178300	-0.52046300
	Ru	1.93404400	-0.00280700	0.80505900
	Ru	-1.93404500	-0.00281100	0.80506200
	C	-2.49226500	-1.30216900	2.06291000

	C	-2.60671500	1.47889900	1.79680900
	O	-3.01184000	2.36814400	2.44071100
	O	-2.81957900	-2.08493900	2.86900400
	N	0.00000000	0.01905900	0.86749100
	O	-4.42706600	-0.30500200	-1.00677500
	C	-1.38101700	-2.82337800	-0.95783100
	C	2.60671700	1.47891700	1.79678400
	O	-2.17612500	-3.63923700	-1.23235300
	O	3.01183600	2.36817300	2.44067400
	C	-3.51312000	-0.18645300	-0.28699700
	Ru	-0.00000600	1.35576400	-0.87964100
	C	-0.00004000	2.89125900	0.22866200
	C	1.48461700	2.01494100	-1.90532400
	C	-1.48454100	2.01497700	-1.90542400
	O	-0.00006100	3.86014300	0.88017200
	O	2.34030900	2.47445700	-2.55447100
	O	-2.34018600	2.47450000	-2.55462700
	N	0.00000000	-0.51449100	-2.06614600
	O	-0.00000300	-0.72076200	-3.25432500

Table S29. The optimized geometry for $Ru_4(N)(NO)(CO)_{10}$ structure **4-NNO-10** (C_s).

mPW1PW91	C	-1.48602300	-2.83490000	0.73730500
	O	-2.33697600	-3.46852700	1.18329600
	C	-0.00037000	-2.81583800	-1.61648400
	O	-0.00047400	-3.39560800	-2.60917000
	C	-3.41987900	-0.21435100	0.24079800
	O	-4.26006800	-0.32996600	1.02315500
	Ru	-0.00019400	-1.83976600	0.04444300
	Ru	-1.87482200	0.03635400	-0.86249200
	Ru	1.87484300	0.03592900	-0.86253500
	C	2.58078500	1.56161600	-1.66356100
	O	2.99216700	2.51311200	-2.17717500
	N	0.00000900	0.02754400	-0.99959200
	O	4.25993500	-0.33065100	1.02324700
	C	1.48547800	-2.83522700	0.73717600
	C	-2.58048500	1.56219300	-1.66347500
	O	2.33633300	-3.46905400	1.18307000
	O	-2.99169600	2.51377600	-2.17706600
	C	3.41980400	-0.21496900	0.24083600
	Ru	0.00018000	1.34876500	0.74676100
	C	0.00033900	2.96540400	-0.34364000
	C	-1.41998800	1.97365200	1.85010400
	C	1.42029800	1.97351900	1.85025100
	O	0.00043300	3.92768400	-0.96261400
	O	-2.26615000	2.34989300	2.52677500
	O	2.26642100	2.34969100	2.52700900
	N	0.00001600	-0.52412000	1.60799400
	O	0.00005000	-0.76372600	2.78616500
BP86	C	-0.78052276	-2.86766039	-1.48692352
	O	-1.24539578	-3.50851633	-2.34721708
	C	1.57783337	-2.90050190	0.00000000
	O	2.56126043	-3.53081280	0.00000000
	C	-0.24255319	-0.16891857	-3.41040050
	O	-1.06006825	-0.26132648	-4.24605223

	Ru	-0.06034073	-1.87694006	0.00000000
	Ru	0.89747092	0.03292694	-1.86379104
	Ru	0.89747092	0.03292694	1.86379104
	C	1.78074415	1.54373967	2.52587757
	O	2.36683709	2.48788158	2.91220844
	N	1.07427909	-0.03964795	0.00000000
	O	-1.06006825	-0.26132648	4.24605223
	C	-0.78052276	-2.86766039	1.48692352
	C	1.78074415	1.54373967	-2.52587757
	O	-1.24539578	-3.50851633	2.34721708
	O	2.36683709	2.48788158	-2.91220844
	C	-0.24255319	-0.16891857	3.41040050
	Ru	-0.78770805	1.38308287	0.00000000
	C	0.30243480	3.00070998	0.00000000
	C	-1.89328423	2.02047667	-1.40791717
	C	-1.89328423	2.02047667	1.40791717
	O	0.90837170	3.99413704	0.00000000
	O	-2.58925834	2.43082276	-2.25007801
	O	-2.58925834	2.43082276	2.25007801
	N	-1.63984786	-0.52277721	0.00000000
	O	-2.83127684	-0.78029332	0.00000000

Table S30. The optimized geometry for $Ru_4(N)(NCO)(CO)_{12}$ structure **4-NNCO-12** (C_{2v}).

mPW1PW91	C	1.48507900	2.45393000	1.43469400
	O	2.33804300	2.98330900	1.99516300
	C	0.00010500	2.93146400	-0.79630800
	O	0.00021300	3.74250700	-1.61157300
	C	3.43621500	0.00002500	0.39665300
	O	4.31344100	0.00008800	1.13990600
	C	2.58024700	1.36506700	-1.89183100
	O	2.98376500	2.18147600	-2.59561800
	Ru	-0.00006400	1.62875400	0.55179100
	Ru	1.90351000	-0.00007400	-0.77134100
	Ru	-1.90332400	-0.00006800	-0.77179900
	C	-2.57978300	1.36507400	-1.89245600
	C	-2.57978200	-1.36540100	-1.89222300
	O	-2.98311700	-2.18192900	-2.59597800
	O	-2.98312600	2.18148200	-2.59634500
	N	0.00010200	-0.00007500	-0.84320300
	O	-4.31375200	0.00009600	1.13882400
	C	-1.48543100	2.45391700	1.43433100
	C	2.58024300	-1.36540900	-1.89159800
	O	-2.33853800	2.98328600	1.99459100
	O	2.98375800	-2.18193900	-2.59524700
	C	-3.43633000	0.00003200	0.39580200
	Ru	-0.00007100	-1.62865000	0.55208600
	C	0.00009200	-2.93160000	-0.79578300
	C	1.48506800	-2.45368100	1.43513300
	C	-1.48543700	-2.45365800	1.43477200
	O	0.00018900	-3.74278700	-1.61090400
	O	2.33802400	-2.98298000	1.99569000
	O	-2.33853800	-2.98293200	1.99513100
	N	-0.00021700	0.00018100	1.96899200
	C	-0.00035600	0.00029000	3.18344200
	O	-0.00049000	0.00039600	4.36127700

BP86	C	-1.48356260	2.49289096	-1.44578808
	O	-2.34033474	3.04552225	-2.01641614
	C	0.00000000	2.96883088	0.78668496
	O	0.00000000	3.80645328	1.60209848
	C	-3.45942915	0.00000000	-0.38945176
	O	-4.34888324	0.00000000	-1.14827450
	C	-2.60649006	1.36322468	1.91687626
	O	-3.02278309	2.18531815	2.63846649
	Ru	0.00000000	1.65575939	-0.55922372
	Ru	-1.92657601	0.00000000	0.78655496
	Ru	1.92657601	0.00000000	0.78655496
	C	2.60649006	1.36322468	1.91687626
	C	2.60649006	-1.36322468	1.91687626
	O	3.02278309	-2.18531815	2.63846649
	O	3.02278309	2.18531815	2.63846649
	N	0.00000000	0.00000000	0.84084711
	O	4.34888324	0.00000000	-1.14827450
	C	1.48356260	2.49289096	-1.44578808
	C	-2.60649006	-1.36322468	1.91687626
	O	2.34033474	3.04552225	-2.01641614
	O	-3.02278309	-2.18531815	2.63846649
	C	3.45942915	0.00000000	-0.38945176
	Ru	0.00000000	-1.65575939	-0.55922372
	C	0.00000000	-2.96883088	0.78668496
	C	-1.48356260	-2.49289096	-1.44578808
	C	1.48356260	-2.49289096	-1.44578808
	O	0.00000000	-3.80645328	1.60209848
	O	-2.34033474	-3.04552225	-2.01641614
	O	2.34033474	-3.04552225	-2.01641614
	N	0.00000000	0.00000000	-1.97578970
	C	0.00000000	0.00000000	-3.20677853
	O	0.00000000	0.00000000	-4.40006337

Table S31. The optimized geometry for $Ru_4(N)(NCO)(CO)_{11}$ structure **4-NNCO-11** (C_s).

mPW1PW91	C	-2.66032082	1.03455903	-1.43947487
	O	-3.35649454	1.42012146	-2.27446244
	C	-0.08713919	0.45242740	-3.46148009
	O	-0.21575156	1.17159169	-4.34998704
	C	-1.13581841	-1.94451474	-2.51532993
	O	-1.89400989	-2.74187216	-2.85651340
	Ru	-1.58135517	0.42398154	0.00007877
	Ru	0.11558354	-0.67334276	-1.91597121
	Ru	0.11555695	-0.67541670	1.91496137
	C	-1.13585160	-1.94723816	2.51293068
	C	1.60810996	-1.63298481	2.60190154
	O	2.49756438	-2.23847704	3.01007982
	O	-1.89404160	-2.74496699	2.85324815
	N	0.13356763	-0.74653613	-0.00054405
	O	-0.21580864	1.16687895	4.35096919
	C	-2.66033726	1.03298879	1.44028512
	C	1.60814401	-1.63016747	-2.60392999
	O	-3.35653199	1.41763077	2.27568002
	O	2.49759137	-2.23524305	-3.01274108
	C	-0.08718648	0.44868016	3.46168289

	Ru	1.50681960	0.91732043	0.00036816
	C	3.00637337	-0.20071637	-0.00022959
	C	2.16066384	1.89398703	-1.50994001
	C	2.16065313	1.89235368	1.51173523
	O	3.93330236	-0.88104828	-0.00058058
	O	2.59382272	2.52036910	-2.37207878
	O	2.59380580	2.51780133	2.37455527
	N	-0.35203291	2.03668890	0.00096252
	C	-0.60615938	3.23021047	0.00159921
	O	-0.81693421	4.38440985	0.00222511
BP86	C	-1.42648300	-2.88184300	0.53548700
	O	-2.26178700	-3.66396900	0.79603100
	C	-3.50001900	-0.22314400	0.25380600
	O	-4.40839900	-0.43764900	0.95864200
	C	-2.52523000	-1.00104700	-2.24157100
	O	-2.86379600	-1.67728900	-3.13602000
	Ru	-0.00001800	-1.70582400	0.09418600
	Ru	-1.94288900	0.11167600	-0.83185500
	Ru	1.94289200	0.11166000	-0.83184000
	C	2.52523900	-1.00104200	-2.24157400
	C	2.62750000	1.70650300	-1.62920000
	O	3.04587900	2.66725400	-2.15011500
	O	2.86381600	-1.67726300	-3.13603300
	N	0.00000300	0.13498800	-0.88245300
	O	4.40844800	-0.43762900	0.95860900
	C	1.42651900	-2.88176900	0.53545600
	C	-2.62753000	1.70650700	-1.62922300
	O	2.26186200	-3.66385600	0.79599800
	O	-3.04594000	2.66724400	-2.15014000
	C	3.50004500	-0.22314100	0.25379700
	Ru	0.00000300	1.31933200	0.95170500
	C	0.00001300	2.95067000	0.02741200
	C	-1.52137200	1.85843200	1.99226000
	C	1.52137100	1.85841400	1.99227900
	O	0.00001300	3.97781900	-0.52994700
	O	-2.39200300	2.23573200	2.67493300
	O	2.39199900	2.23570300	2.67496200
	N	-0.00001400	-0.67235600	1.84888100
	C	-0.00002400	-1.08761900	3.01395800
	O	-0.00003900	-1.44423400	4.14821600

Table S32. The optimized geometry for $Ru_4(N)(NCO)(CO)_{10}$ structure **4-NNCO-10** (C_s).

mPW1PW91	C	-1.53614400	-2.81785400	0.42800600
	O	-2.40976600	-3.47661300	0.78740100
	C	-0.00012900	-2.68459700	-1.80787300
	O	-0.00017300	-3.22687700	-2.82464500
	C	-3.42656200	-0.21116800	0.16445200
	O	-4.26820800	-0.37640600	0.93624900
	Ru	-0.00006700	-1.81656200	-0.15111300
	Ru	-1.88349300	0.11325400	-0.91818800
	Ru	1.88350100	0.11310900	-0.91820800
	C	2.60032300	1.67988000	-1.62058400
	O	3.02426800	2.65533700	-2.07611300
	N	0.00000300	0.09935300	-1.04018000

	O	4.26816900	-0.37660800	0.93627100
	C	1.53598600	-2.81793100	0.42794200
	C	-2.60023100	1.68008000	-1.62052900
	O	2.40958400	-3.47674600	0.78729300
	O	-3.02412000	2.65557100	-2.07603900
	C	3.42654300	-0.21135400	0.16445600
	Ru	0.00006100	1.32801400	0.73857900
	C	0.00011200	2.92933200	-0.26147800
	C	-1.45436200	1.91832500	1.82700800
	C	1.45446200	1.91828100	1.82706400
	O	0.00014100	3.91215700	-0.85131600
	O	-2.31774300	2.27727600	2.48988700
	O	2.31782400	2.27721100	2.48997900
	N	-0.00000100	-0.61127100	1.66094500
	C	0.00000200	-0.94991000	2.82911800
	O	0.00000800	-1.27917000	3.95879100
BP86	C	-0.48563664	-2.84740550	-1.54541340
	O	-0.86685150	-3.50693079	-2.43363127
	C	1.74861618	-2.81596296	0.00000000
	O	2.74923321	-3.42391891	0.00000000
	C	-0.18826055	-0.15905643	-3.40502029
	O	-1.00607445	-0.29013647	-4.23536276
	Ru	0.12845313	-1.86803504	0.00000000
	Ru	0.95173226	0.09941400	-1.87005540
	Ru	0.95173226	0.09941400	1.87005540
	C	1.74305467	1.65662624	2.53818220
	O	2.27617758	2.62751818	2.93559059
	N	1.12466204	0.01626900	0.00000000
	O	-1.00607445	-0.29013647	4.23536276
	C	-0.48563664	-2.84740550	1.54541340
	C	1.74305467	1.65662624	-2.53818220
	O	-0.86685150	-3.50693079	2.43363127
	O	2.27617758	2.62751818	-2.93559059
	C	-0.18826055	-0.15905643	3.40502029
	Ru	-0.76717302	1.37356591	0.00000000
	C	0.24241790	2.97392296	0.00000000
	C	-1.85070788	1.98789394	-1.43886581
	C	-1.85070788	1.98789394	1.43886581
	O	0.83522885	3.97766199	0.00000000
	O	-2.53286280	2.38953879	-2.29541882
	O	-2.53286280	2.38953879	2.29541882
	N	-1.66252693	-0.58897013	0.00000000
	C	-2.84938291	-0.92584619	0.00000000
	O	-3.99460990	-1.25604524	0.00000000

Table S33. The optimized geometry for $Ru_4(N)_2(CO)_{12}$ structure **4-NN-12** (C_s).

mPW1PW91	C	-0.15418700	-2.07123000	-2.69804900
	O	-0.41374500	-2.99192600	-3.33172200
	C	-0.15419600	0.65559900	-3.10873200
	O	-0.46648600	1.35441200	-3.96750900
	C	-2.53090100	-2.49446900	0.00000000
	O	-2.86696600	-3.59223800	0.00000000
	C	-3.00022500	-0.12013500	-1.41654000
	O	-3.68087400	0.26137300	-2.26218600

	Ru	0.28082400	-0.55166600	-1.63279800
	Ru	-1.86888400	-0.69626200	0.00000000
	Ru	1.13738100	1.68493300	0.00000000
	C	1.11756500	2.99846000	-1.36007900
	C	1.11756500	2.99846000	1.36007900
	O	1.06623100	3.81131400	2.17041800
	O	1.06623100	3.81131400	-2.17041800
	N	-0.42846000	0.68510300	0.00000000
	O	4.21643800	1.53454500	0.00000000
	C	2.13572100	-0.69930100	-1.97537200
	C	-3.00022500	-0.12013500	1.41654000
	O	3.25127400	-0.89093600	-2.19179600
	O	-3.68087400	0.26137300	2.26218600
	C	3.07253500	1.62479800	0.00000000
	Ru	0.28082400	-0.55166600	1.63279800
	C	-0.15419600	0.65559900	3.10873200
	C	-0.15418700	-2.07123000	2.69804900
	C	2.13572100	-0.69930100	1.97537200
	O	-0.46648600	1.35441200	3.96750900
	O	-0.41374500	-2.99192600	3.33172200
	O	3.25127400	-0.89093600	2.19179600
	N	0.14124000	-1.70571500	0.00000000
BP86	C	-1.41067500	2.77483300	-1.48876300
	O	-2.18328100	3.47226600	-2.01652900
	C	0.31995400	3.13411100	0.59880800
	O	0.53070500	3.99720300	1.35940100
	C	-3.58537100	-0.00005600	-0.33425300
	O	-4.56124700	-0.00009200	-0.97383900
	C	-2.44083900	1.41734100	1.80112800
	O	-2.73393300	2.27259400	2.54283200
	Ru	-0.12838400	1.65937400	-0.61374500
	Ru	-1.92227000	0.00000300	0.63004800
	Ru	1.96663600	-0.00001400	0.59984300
	C	2.78058200	1.36239600	1.64032500
	C	2.78048900	-1.36236500	1.64046200
	O	3.26668700	-2.18034000	2.31852800
	O	3.26683700	2.18041500	2.31829500
	N	0.09242500	-0.00000200	0.78968900
	O	4.27270300	-0.00020400	-1.47990900
	C	1.21146800	1.98580500	-1.92388500
	C	-2.44081500	-1.41721100	1.80129500
	O	1.95533200	2.20458800	-2.80133200
	O	-2.73387600	-2.27239700	2.54308900
	C	3.42914800	-0.00012400	-0.67367500
	Ru	-0.12839800	-1.65936600	-0.61375700
	C	0.31996800	-3.13410800	0.59877300
	C	-1.41080100	-2.77477300	-1.48866800
	C	1.21135500	-1.98584900	-1.92399300
	O	0.53074900	-3.99720300	1.35935600
	O	-2.18348100	-3.47217500	-2.01637000
	O	1.95515300	-2.20465200	-2.80148900
	N	-0.99231700	0.00001700	-1.41922100

Table S34. The optimized geometry for $Ru_4(N)_2(CO)_{11}$ structure **4-NN-11**(C_s).

mPW1PW91	C	-1.36894100	-2.75933600	1.19514800
	O	-2.15125700	-3.48533400	1.62516000
	C	-3.48012300	-0.22204900	0.34466800
	O	-4.37917700	-0.34921500	1.05049700
	C	-2.47606500	-1.28493500	-2.03384900
	O	-2.80209300	-2.03012500	-2.84862800
	Ru	-0.00002400	-1.61380300	0.51423500
	Ru	-1.90693300	-0.02688200	-0.74540300
	Ru	1.90693000	-0.02690400	-0.74539800
	C	2.47606100	-1.28493200	-2.03387700
	C	2.58013200	1.48946600	-1.66669600
	O	2.97323500	2.39179900	-2.26289800
	O	2.80209300	-2.03010000	-2.84867400
	N	0.00000000	0.00440500	-0.83813900
	O	4.37916600	-0.34924100	1.05051500
	C	1.36895700	-2.75930900	1.19506800
	C	-2.58011700	1.48948200	-1.66673600
	O	2.15131000	-3.48529100	1.62503900
	O	-2.97321200	2.39180500	-2.26295900
	C	3.48011700	-0.22207600	0.34468000
	Ru	0.00001300	1.29371200	0.93746400
	C	0.00002900	2.91097100	-0.18249600
	C	-1.44702400	1.92261100	2.00853600
	C	1.44700400	1.92262100	2.00859100
	O	0.00003500	3.83495400	-0.86192500
	O	-2.27979500	2.32779200	2.69018600
	O	2.27975200	2.32780200	2.69027000
	N	-0.00000100	-0.45057500	1.90522500
BP86	C	-1.35387200	-2.74438400	1.26871200
	O	-2.12211900	-3.54628400	1.63923500
	C	-3.51530200	-0.21651800	0.32082700
	O	-4.43048600	-0.31887900	1.04121100
	C	-2.48028900	-1.38767400	-2.00553900
	O	-2.80770000	-2.17956400	-2.80287200
	Ru	0.00001200	-1.54220900	0.64258300
	Ru	-1.93029000	-0.06027700	-0.76795700
	Ru	1.93028200	-0.06024600	-0.76796100
	C	2.48029200	-1.38762800	-2.00555100
	C	2.60293100	1.41789700	-1.76426500
	O	3.00324500	2.30130300	-2.41893100
	O	2.80770700	-2.17950900	-2.80289100
	N	-0.00000400	-0.02325800	-0.83723900
	O	4.43051500	-0.31883300	1.04116100
	C	1.35396200	-2.74430600	1.26873100
	C	-2.60297200	1.41785700	-1.76424900
	O	2.12225000	-3.54614900	1.63929100
	O	-3.00330700	2.30125600	-2.41891000
	C	3.51531400	-0.21647500	0.32079900
	Ru	-0.00002300	1.33155200	0.93414300
	C	-0.00004200	2.92170100	-0.24773600
	C	-1.45938600	2.02117200	1.96996100
	C	1.45941800	2.02111100	1.96989400
	O	-0.00006100	3.84972200	-0.95205000

O	-2.30255500	2.48664300	2.63095700
O	2.30263100	2.48655400	2.63085600
N	-0.00006300	-0.36741300	2.03702300

Table S35. The optimized geometry for $Ru_4(N)_2(CO)_{10}$ structure **4-NN-10-1**(C_{2v}).

mPW1PW91	C	1.43303217	-2.36707872	-1.53041309
	O	2.24757141	-3.01994460	-2.01458232
	C	3.51728029	0.00000000	-0.29638467
	O	4.43796294	0.00000000	-0.98634394
	C	2.49836460	-1.41201870	1.88828308
	O	2.82962019	-2.25138219	2.60292564
	Ru	0.00000000	-1.34525542	-0.76772699
	Ru	1.91833189	0.00000000	0.76759432
	Ru	-1.91833189	0.00000000	0.76759432
	C	-2.49836460	-1.41201870	1.88828308
	C	-2.49836460	1.41201870	1.88828308
	O	-2.82962019	2.25138219	2.60292564
	O	-2.82962019	-2.25138219	2.60292564
	N	0.00000000	0.00000000	0.86418064
	O	-4.43796294	0.00000000	-0.98634394
	C	-1.43303217	-2.36707872	-1.53041309
	C	2.49836460	1.41201870	1.88828308
	O	-2.24757141	-3.01994460	-2.01458232
	O	2.82962019	2.25138219	2.60292564
	C	-3.51728029	0.00000000	-0.29638467
	Ru	0.00000000	1.34525542	-0.76772699
	C	1.43303217	2.36707872	-1.53041309
	C	-1.43303217	2.36707872	-1.53041309
	O	2.24757141	3.01994460	-2.01458232
	O	-2.24757141	3.01994460	-2.01458232
	N	0.00000000	0.00000000	-2.01488616
BP86	C	1.44157439	-2.42783243	-1.49191332
	O	2.26301348	-3.14243326	-1.91928843
	C	3.55444010	0.00000000	-0.28686556
	O	4.49347816	0.00000000	-0.98394548
	C	2.51040203	-1.41916217	1.89700701
	O	2.84276503	-2.26987432	2.62877183
	Ru	0.00000000	-1.33830970	-0.81801718
	Ru	1.94171401	0.00000000	0.76793831
	Ru	-1.94171401	0.00000000	0.76793831
	C	-2.51040203	-1.41916217	1.89700701
	C	-2.51040203	1.41916217	1.89700701
	O	-2.84276503	2.26987432	2.62877183
	O	-2.84276503	-2.26987432	2.62877183
	N	0.00000000	0.00000000	0.84180115
	O	-4.49347816	0.00000000	-0.98394548
	C	-1.44157439	-2.42783243	-1.49191332
	C	2.51040203	1.41916217	1.89700701
	O	-2.26301348	-3.14243326	-1.91928843
	O	2.84276503	2.26987432	2.62877183
	C	-3.55444010	0.00000000	-0.28686556
	Ru	0.00000000	1.33830970	-0.81801718
	C	1.44157439	2.42783243	-1.49191332
	C	-1.44157439	2.42783243	-1.49191332
	O	2.26301348	3.14243326	-1.91928843
	O	-2.26301348	3.14243326	-1.91928843
	N	0.00000000	0.00000000	-2.10140785

Table S36. The optimized geometry for $Ru_4(N)_2(CO)_{10}$ structure **4-NN-10-2** (C_s).

mPW1PW91	C	-2.78433011	1.32324442	-1.40421214
	O	-3.12470034	2.01891727	-2.25248507
	C	-3.72879497	-0.88254323	0.00000000
	O	-4.63795491	-1.58126266	0.00000000
	C	-0.05188706	-0.06838082	-3.05092538
	O	-0.16422598	0.40143188	-4.09885644
	Ru	-2.14791314	0.21364437	0.00000000
	Ru	-0.00702404	-0.86712651	-1.32131041
	Ru	-0.00702404	-0.86712651	1.32131041
	C	1.45188439	-2.00060605	1.81353926
	O	2.35406734	-2.66631181	2.07606195
	N	-1.08478661	-1.72847260	0.00000000
	O	-0.16422598	0.40143188	4.09885644
	C	-2.78433011	1.32324442	1.40421214
	C	1.45188439	-2.00060605	-1.81353926
	O	-3.12470034	2.01891727	2.25248507
	O	2.35406734	-2.66631181	-2.07606195
	C	-0.05188706	-0.06838082	3.05092538
	Ru	1.70877230	0.87729641	0.00000000
	C	3.44568339	0.06737413	0.00000000
	C	2.13684157	2.06310516	-1.40210909
	C	2.13684157	2.06310516	1.40210909
	O	4.46286758	-0.46696088	0.00000000
	O	2.37943548	2.81106982	-2.24158065
	O	2.37943548	2.81106982	2.24158065
	N	-0.21652834	0.68937816	0.00000000
BP86	C	-2.79776192	1.34118618	-1.41146924
	O	-3.14812645	2.04983198	-2.27088182
	C	-3.75848272	-0.84762574	0.00000000
	O	-4.69808555	-1.53810838	0.00000000
	C	-0.12268963	-0.12257531	-3.09505280
	O	-0.28903998	0.31293035	-4.17108539
	Ru	-2.15971027	0.22838526	0.00000000
	Ru	0.00704127	-0.86952849	-1.33641314
	Ru	0.00704127	-0.86952849	1.33641314
	C	1.43573100	-2.03502210	1.85444125
	O	2.32265647	-2.74258028	2.14247682
	N	-1.08010352	-1.73379658	0.00000000
	O	-0.28903998	0.31293035	4.17108539
	C	-2.79776192	1.34118618	1.41146924
	C	1.43573100	-2.03502210	-1.85444125
	O	-3.14812645	2.04983198	2.27088182
	O	2.32265647	-2.74258028	-2.14247682
	C	-0.12268963	-0.12257531	3.09505280
	Ru	1.74684701	0.90749391	0.00000000
	C	3.47217454	0.06800837	0.00000000
	C	2.19034031	2.08605680	-1.41428176
	C	2.19034031	2.08605680	1.41428176
	O	4.49610768	-0.49561824	0.00000000
	O	2.45270064	2.84336034	-2.26683562
	O	2.45270064	2.84336034	2.26683562
	N	-0.19671799	0.71147842	0.00000000

Table S37. Total energies with ZPVE correction (E in Hartree), relative energies (ΔE in kcal/mol), Total free energies with ZPVE correction (G in Hartree), and relative free energies (ΔG in kcal/mol). Neither structure has any imaginary vibrational frequencies.

		**10S-1**(C_{2v})	**10S-2**(C_s)
mPW1PW91	E	-1678.09676	-1678.06751
	ΔE	0.0	18.4
	G	-1678.16135	-1678.13127
	ΔG	0.0	18.9
BP86	E	-1678.83658	-1678.80620
	ΔE	0.0	19.1
	G	-1678.90334	-1678.87192
	ΔG	0.0	19.7

Table S38. Total energies with ZPVE correction (E in Hartree), Total free energies with ZPVE correction (G in Hartree), number of imaginary vibrational frequencies (Nimg) for the $Ru_3(NO)_2(CO)_n$ (n = 9, 8, 7) structures.

		**9S-1**(C_s)	**8S-1**(C_{2v})	**7S-1**(C_s)
mPW1PW91	E	-1564.76049	-1451.35378	-1338.05385
	G	-1564.82262	-1451.41331	-1338.11144
	Nimg	0	0	0
BP86	E	-1565.46717	-1452.04220	-1338.69813
	G	-1565.53126	-1452.10147	-1338.75604
	Nimg	0	1(56i)	1(28i)

Table S39. Total energies with ZPVE correction (E in Hartree), total free energies with ZPVE correction (G in Hartree), and number of imaginary vibrational frequencies (Nimg) for the $Ru_3(CO)_n(N)(NO)$ (n = 10, 9, 8) structures. None of the structures exhibited any imaginary vibrational frequencies.

		**3-NNO-10** (C_1)	**3-NNO-9-1** (C_s)	**3-NNO-9** (C_s)	**3-NNO-8** (C_s)
mPW1PW91	E	-1602.86190	-1489.54144	-1489.50434	-1376.17104
	ΔE	—	0.0	23.3	0.0
	G	-1602.92684	-1489.60388	-1489.56515	-1376.23073
	ΔG	—	0.0	24.3	0.0
BP86	E	-1603.57736	-1490.22939	-1490.18882	-1376.82846
	ΔE	—	0.0	25.4	0.0
	G	-1603.64429	-1490.29366	-1490.25144	-1376.89011
	ΔG	—	0.0	26.5	0.0

Table S40. Total energies with ZPVE correction (E in Hartree), Total free energies with ZPVE correction (G in Hartree), number of imaginary vibrational frequencies (Nimg) for the $Ru_3(N)_2(CO)_n$ (*n* = 10, 9, 8) structures.

		**3-NN-10**(C_s)	**3-NN-9**(C_s)	**3-NN-8**(C_s)
mPW1PW91	E	-1527.67100	-1414.36221	-1301.00423
	G	-1527.73328	-1414.42119	-1301.06259
	Nimg	2(36i,3i)	1(2i)	0
BP86	E	-1528.35660	-1415.01736	-1301.62441
	G	-1528.42520	-1415.08228	-1301.68421
	Nimg	1(34i)	0	0

Table S41. Total energies with ZPVE correction (E in Hartree), Total free energies with ZPVE correction (G in Hartree), number of imaginary vibrational frequencies (Nimg) for the $Ru_3(N)(NCO)(CO)_n$ (*n* = 9,8,7) structures.

		**3-NNCO-9**(C_s)	**3-NNCO-9-1**(C_s)	**3-NNCO-8** (C_s)	**3-NNCO-7** (C_1)
mPW1PW91	E	-1527.71584	-1527.71794	-1414.34869	-1301.00896
	ΔE	0.0	-1.3	—	—
	G	-1527.78049	-1527.78116	-1414.40706	-1301.06878
	ΔG	0.0	-0.4	—	—
	Nimg	0	0	1(46i)	0
BP86	E	-1528.40107	-1528.39598	-1414.99862	-1301.63219
	ΔE	0.0	3.2	—	—
	G	-1528.46735	-1528.46025	-1415.05845	-1301.69392
	ΔG	0.0	4.6	—	—
	Nimg	0	0	1(42i)	0

Table S42. Wiberg bond indices (WBI) for the Ru–Ru bond sand natural charges on the ruthenium atoms for of all the structures.

	Method	WBI of Ru–Ru bond	Ru–Ru bond distance	Ru–Ru bond order	Natural charges on Ru
**3-$(NO)_2$-10-1**	mPW1PW91	0.12	3.178	0	-1.136,1.136, -1.636,
		0.32	2.888	1	
	BP86	0.12	3.226	0	-1.090,1.090, -1.541,
		0.29	2.953	1	
**3-$(NO)_2$-10-2**	mPW1PW91	0.07	3.306	0	-1.021, -1.021, -1.467,
		0.35	2.806	1	
		0.35	2.092	1	
	BP86	0.08	3.356	0	-0.945, -0.945, -1.338
		0.32	2.853	1	
		0.32	2.131	1	
**3-$(NO)_2$-9**	mPW1PW91	0.07	3.296	0	-1.067, -1.067,-1.137
		0.37	2.780	1	
	BP86	0.08	3.353	0	-0.981, -0.981, -1.028
		0.33	2.829	1	
**3-$(NO)_2$-8**	mPW1PW91	0.07	3.382	0	-1.187, -1.187, -0.126
		0.21	2.681	1	
	BP86	0.06	3.490	0	-1.101, -1.101, -0.113
		0.21	2.786	1	
**3-$(NO)_2$-7**	mPW1PW91	0.17	3.058	0	-0.394, -0.394, -1.235
		0.39	2.730	1	
	BP86	0.22	3.047	0	-0.367, -0.367, -1.120,
		0.36	2.763	1	
**3-NNO-10**	mPW1PW91	0.36	2.750	1	-0.758, -1.160, -1.490
		0.40	2.699	1	
	BP86	0.26	2.782	1	-0.795, -1.196, -1.196
		0.26	2.723	1	
**3-NNO-9-1**	mPW1PW91	0.36	2.786	1	-1.056, -1.078, -1.078
	BP86	0.33	2.836	1	-0.966, -0.987, -0.987
**3-NNO-9-2**	mPW1PW91	0.06	3.307	0	-1.138, -1.012, -1.012
		0.34	2.704	1	
	BP86	0.07	3.372	0	-1.043, -0.926, -0.926
		0.31	2.751	1	
**3-NNO-8**	mPW1PW91	0.07	3.316	0	-1.184,-1.184, -0.104
		0.30	2.754	1	
	BP86	0.08	3.371	0	-1.086, -0.096, -0.096
		0.30	2.798	1	
**3-NN-10**	mPW1PW91	0.03	4.342	0	-0.981, -0.981, -1.318
		0.31	2.911	1	
	BP86	0.02	4.483		-0.915, -0.915, -1.194
		0.31	2.950	1	
**3-NN-9**	mPW1PW91	0.35	2.805	1	-1.163, -0.910, -0.910
	BP86	0.32	2.858	1	-0.839, -0.839-1.038
**3-NN-8**	mPW1PW91	0.37	2.776	1	-0.440, -0.976, -0.976
	BP86	0.34	2.824	1	-0.378, -0.895, 0.895
**3-NNCO-9-1**	mPW1PW91	0.07	3.274	0	-0.998, -0.998, -1.046
		0.36	2.787	1	
	BP86	0.14	3.321	0	-0.923, -0.923, -0.957
		0.34	2.839	1	
**3-NNCO-9-2**	mPW1PW91	0.08	3.341	0	-0.995, -0.995, -0.674,

		0.40	2.782	1	
	BP86	0.08	3.384	0	-0.648, -0.956, -0.956
		0.38	2.833	1	
**3-NNCO-8**	mPW1PW91	0.06	3.305	0	-1.107, -1.107, -0.093
		0.30	2.754	1	
	BP86	0.06	3.349	0	-1.025, -1.025, -0.083
		0.31	2.798	1	
**3-NNCO-7**	mPW1PW91	0.21	2.838	1	-0.684, -0.222, -0.640
		0.34	2.704	1	
	BP86	0.21	2.855	1	-0.639, -0.254, -0.544
		0.34	2.756	1	
**3-NONCO-10**	mPW1PW91	0.12	3.111	0	-1.086, -1.086, -1.629
		0.35	2.866	1	
	BP86	0.12	3.147	0	-1.008, -1.008, -1.493
		0.31	2.925	1	
**3-NONCO-9**	mPW1PW91	0.07	3.302	0	-1.070, -1.070, -1.103
		0.42	2.738	1	
	BP86	0.16	3.356	0	-0.982, -0.982, -1.011
		0.48	2.788	1	
**4-NNO-12**	mPW1PW91	0.31	2.837	1	-1.205, -1.031, -1.031, -1.205
	BP86	0.28	2.878	1	-1.101, -0.941, -0.941, -1.101
**4-NNO-11**	mPW1PW91	0.33	2.792	1	-0.600, -1.071, -1.071, -1.230
		0.30	2.857	1	
	BP86	0.31	2.827	1	-0.580, -0.972, -0.972,-1.131,
		0.28	2.902	1	
**4-NNO-10**	mPW1PW91	0.30	2.803	1	-1.330, -0.358, -0.358, -1.330
	BP86	0.30	2.805	1	-1.228, -0.330, -0.330, -1.009
**4-NNCO-12**	mPW1PW91	0.31	2.833	1	-1.140, -1.016, -1.016, -1.140
	BP86	0.29	2.875	1	-1.048, -0.929, -0.929, -1.048
**4-NNCO-11**	mPW1PW91	0.34	2.853	1	-0.433, -1.066, -1.066, -1.150
	BP86	0.32	2.901	1	-0.414, -0.974, -0.974, -1.057
**4-NNCO-10**	mPW1PW91	0.42	2.787	1	-1.250, -0.341, -0.341, -1.059
	BP86	0.40	2.842	1	-1.161, -0.313, -0.313, -0.958
**4-NN-12**	mPW1PW91	0.31	2.703	1	-1.111, -1.128, -0.915, -1.111
	BP86	0.28	2.742	1	-1.018, -1.020, -0.840, -1.018
**4-NN-11**	mPW1PW91	0.33	2.783	1	-0.518, -1.065, -1.065, -1.223
	BP86	0.31	2.813	1	-0.476, -0.970, -0.970, -1.118
**4-NN-10-1**	mPW1PW91	0.33	2.801	1	-0.590, -1.109, -1.109, -0.590
	BP86	0.31	2.842	1	-0.538, -1.008, -1.008, -0.538
**4-NN-10-2**	mPW1PW91	0.37	2.738	1	-1.035, -0.636, -0.636, -0.983
	BP86	0.35	2.772	1	-0.955, -0.569, -0.569, -0.913

Complete Gaussian 09 reference (Reference 16)

M.J. Frisch, G.W. Trucks, H.B. Schlegel, G.E. Scuseria, M.A. Robb, J.R. Cheeseman, G. Scalmani, V. Barone, B. Mennucci, G.A. Petersson, H. Nakatsuji, M. Caricato, X. Li, H.P. Hratchian, A.F. Izmaylov, J. Bloino, G. Zheng, J.L. Sonnenberg, M. Hada, M. Ehara, K. Toyota, R. Fukuda, J. Hasegawa, M. Ishida, T. Nakajima, Y. Honda, O. Kitao, H. Nakai, T. Vreven, J.A. Montgomery, J.E. Peralta, F. Ogliaro, M. Bearpark, J.J. Heyd, E. Brothers, K.N. Kudin, V.N. Staroverov, T. Keith, R. Kobayashi, J. Normand, K. Raghavachari, A. Rendell, J.C. Burant, S.S. Iyengar, J. Tomasi, M. Cossi, N. Rega, J.M. Millam, M. Klene, J.E. Knox, J.B. Cross, V. Bakken, C. Adamo, J. Jaramillo, R. Gomperts, R.E. Stratmann, O. Yazyev, A.J. Austin, R. Cammi, C. Pomelli, J.W. Ochterski, R.L. Martin, K. Morokuma, V.G. Zakrzewski, G.A. Voth, P. Salvador, J.J. Dannenberg, S. Dapprich, A.D. Daniels, O. Farkas, J.B. Foresman, J.V. Ortiz, J. Cioslowski, and D.J. Fox, Gaussian 09, Revision A.02; Gaussian, Inc., Wallingford CT, 2009.
